# Supplementary material for: Mark-release-recapture of male Aedes aegypti (Diptera: Culicidae): Use of rhodamine B to estimate movement, mating and population parameters in preparation for an incompatible male program
Source: PLoS Negl Trop Dis. 2021 Jun 7;15(6):e0009357. doi: 10.1371/journal.pntd.0009357 (PMC8183986; doi:10.1371/journal.pntd.0009357)

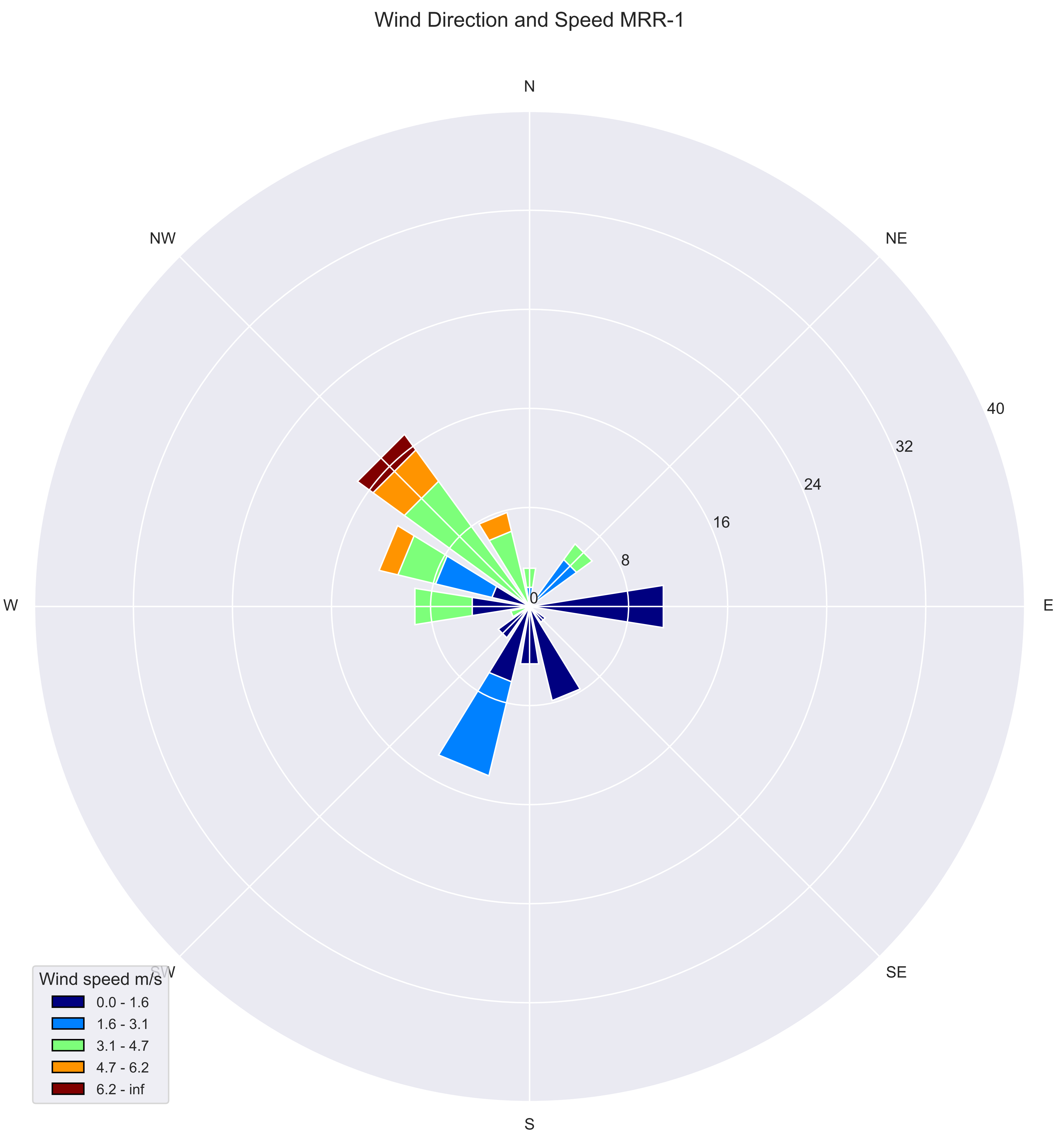

Wind Direction and Speed MRR-1
N
NE
E
SE
S
SW
W
NW
0
8
16
24
32
40
Wind speed m/s
0.0 - 1.6
1.6 - 3.1
3.1 - 4.7
4.7 - 6.2
6.2 - inf

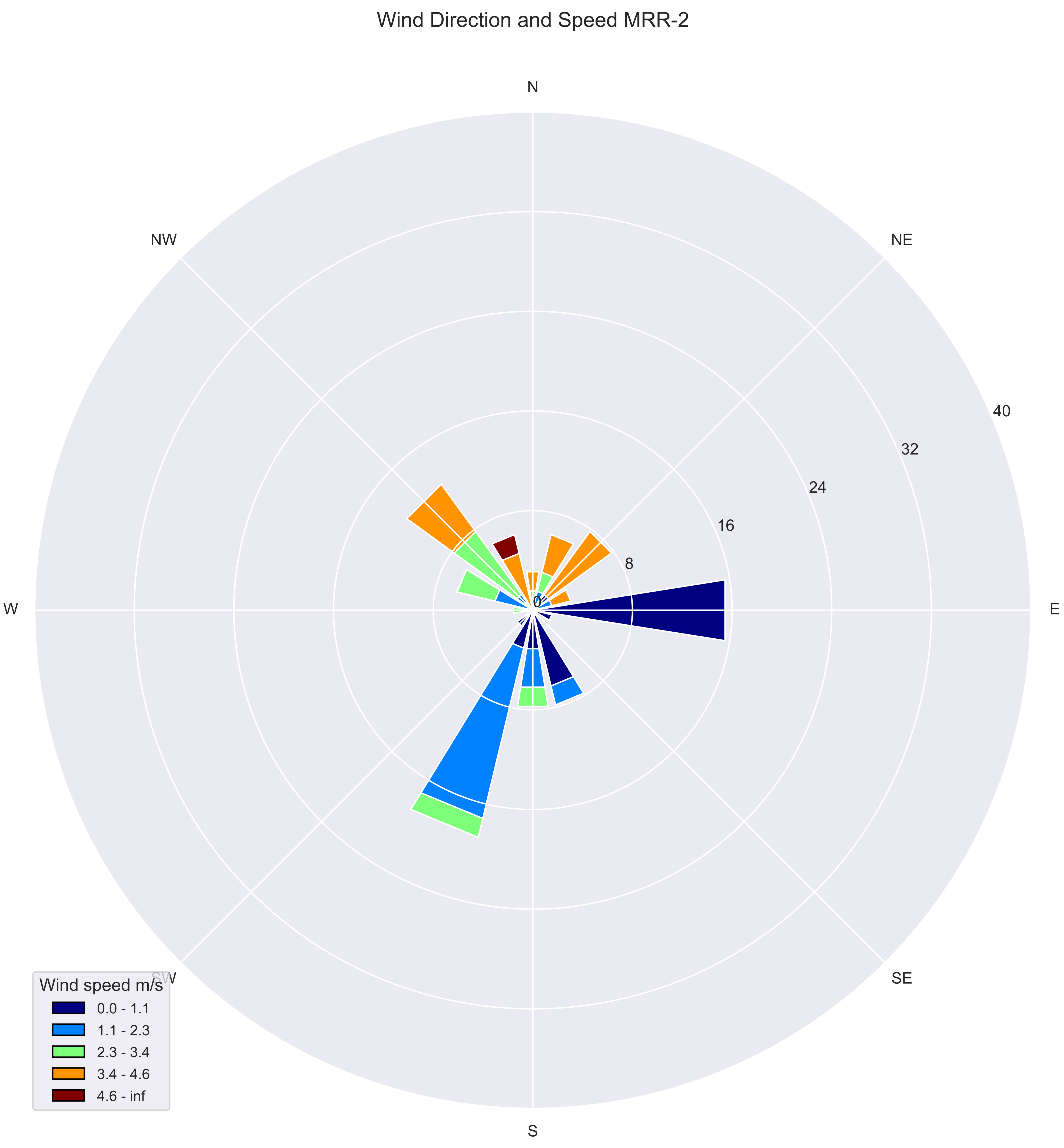
Wind Direction and Speed MRR-2
N
NE
E
SE
S
SW
W
NW
0
8
16
24
32
40
Wind speed m/s
0.0 - 1.1
1.1 - 2.3
2.3 - 3.4
3.4 - 4.6
4.6 - inf

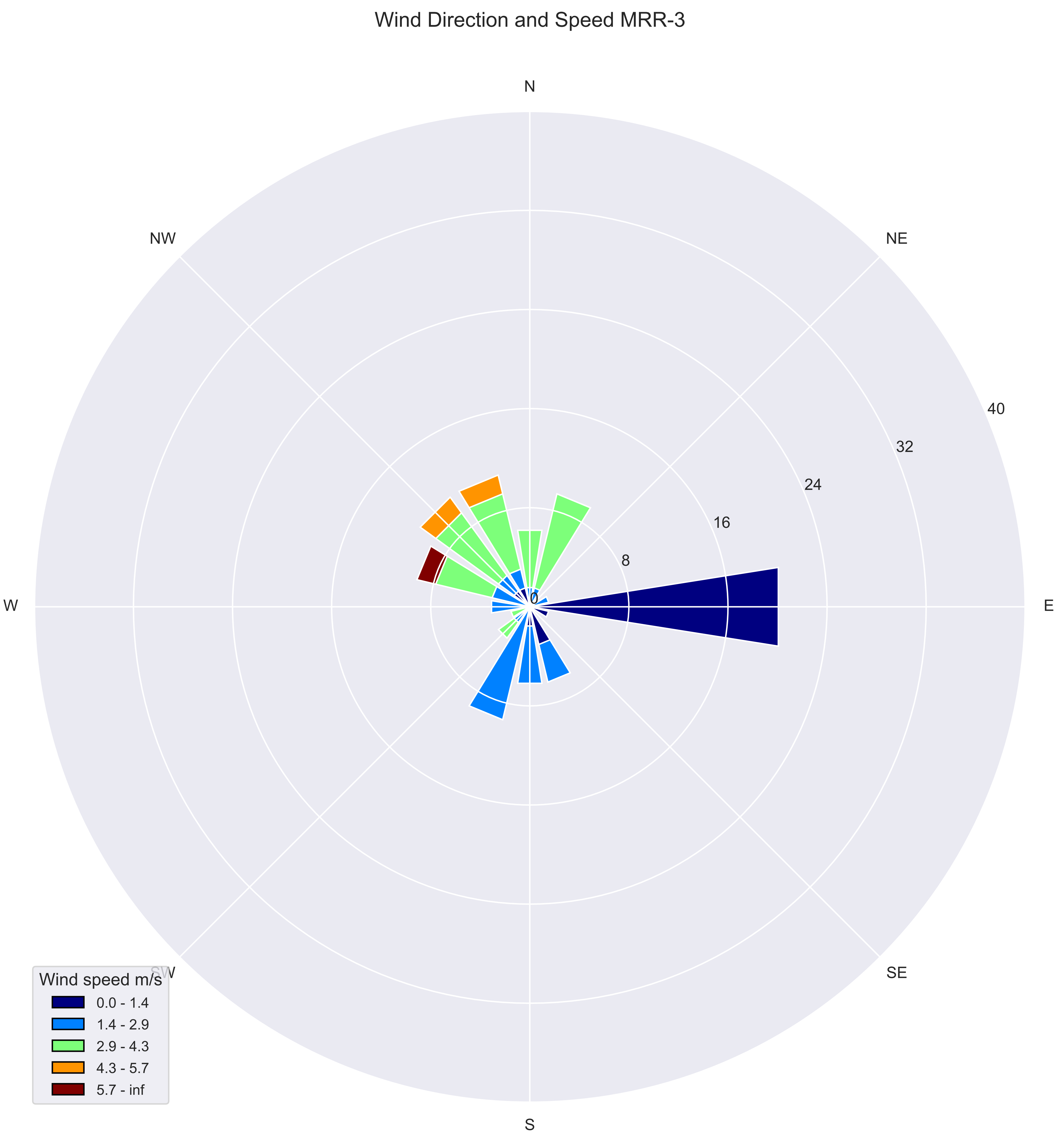

Wind Direction and Speed MRR-3
N
NE
E
SE
S
SW
W
NW
0
8
16
24
32
40
Wind speed m/s
0.0 - 1.4
1.4 - 2.9
2.9 - 4.3
4.3 - 5.7
5.7 - inf

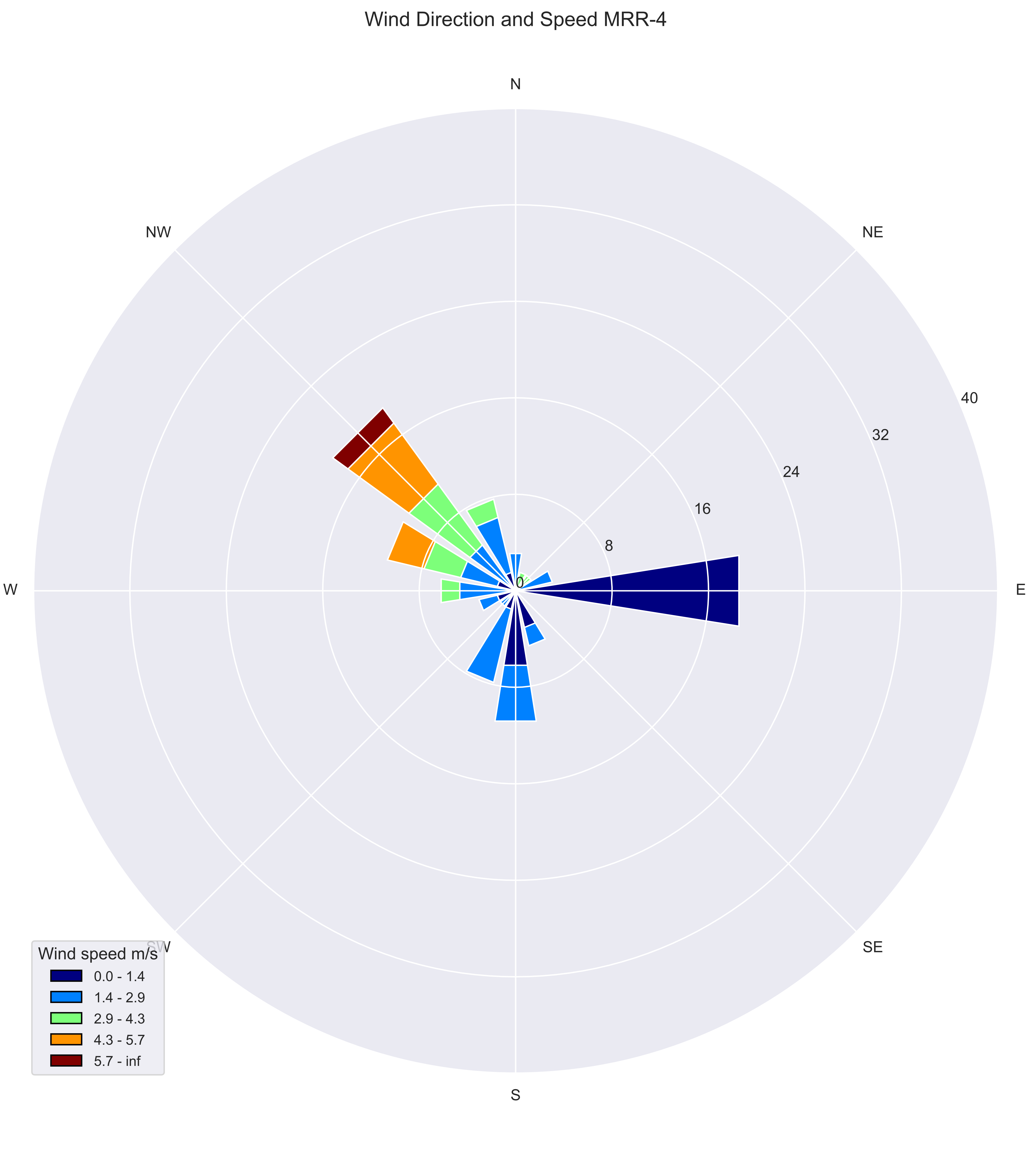

Wind Direction and Speed MRR-4
N
NE
E
SE
S
SW
W
NW
0
8
16
24
32
40
Wind speed m/s
0.0 - 1.4
1.4 - 2.9
2.9 - 4.3
4.3 - 5.7
5.7 - inf

Wind Direction and Speed MRR-5

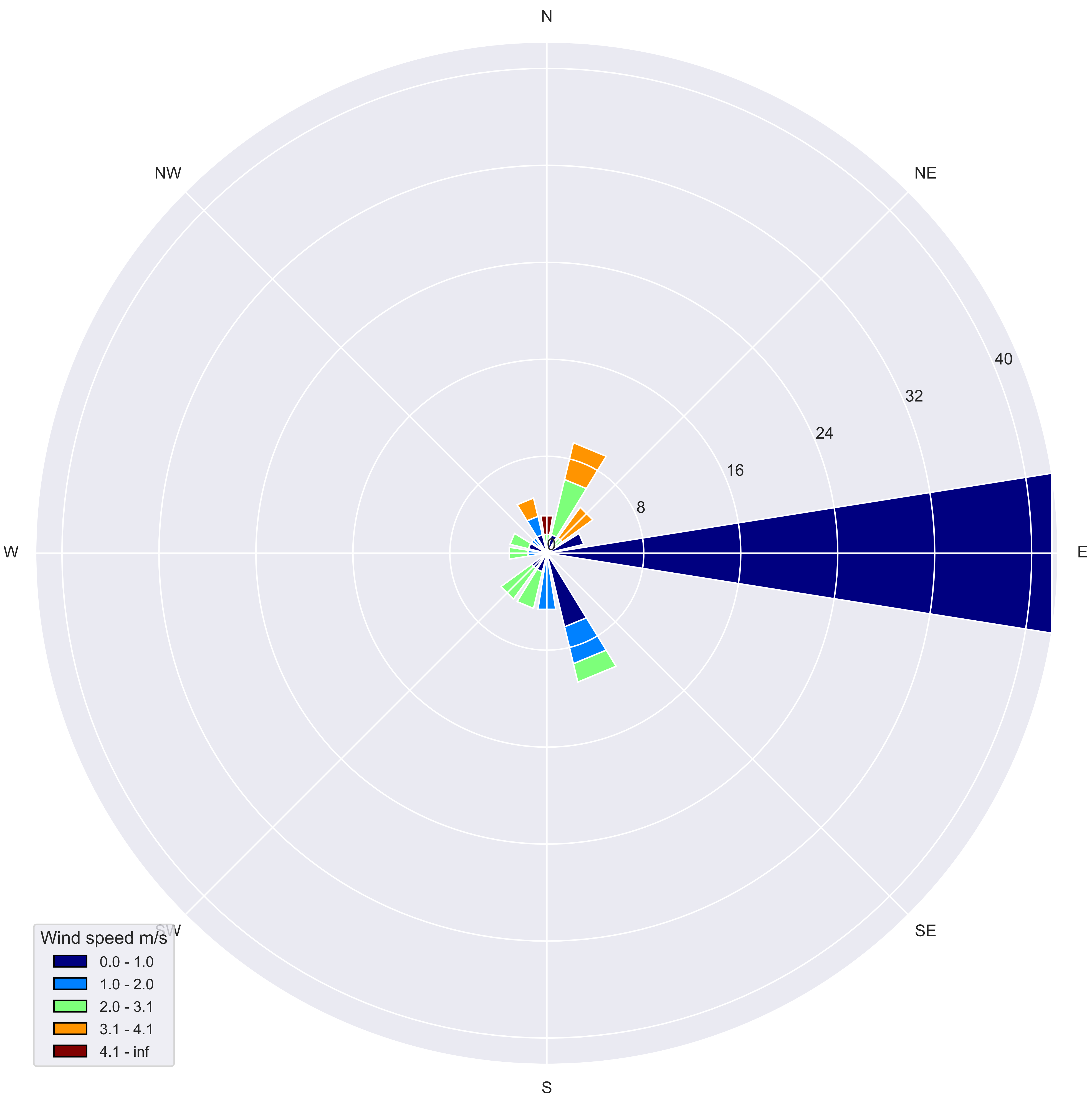

Wind Direction and Speed MRR-6

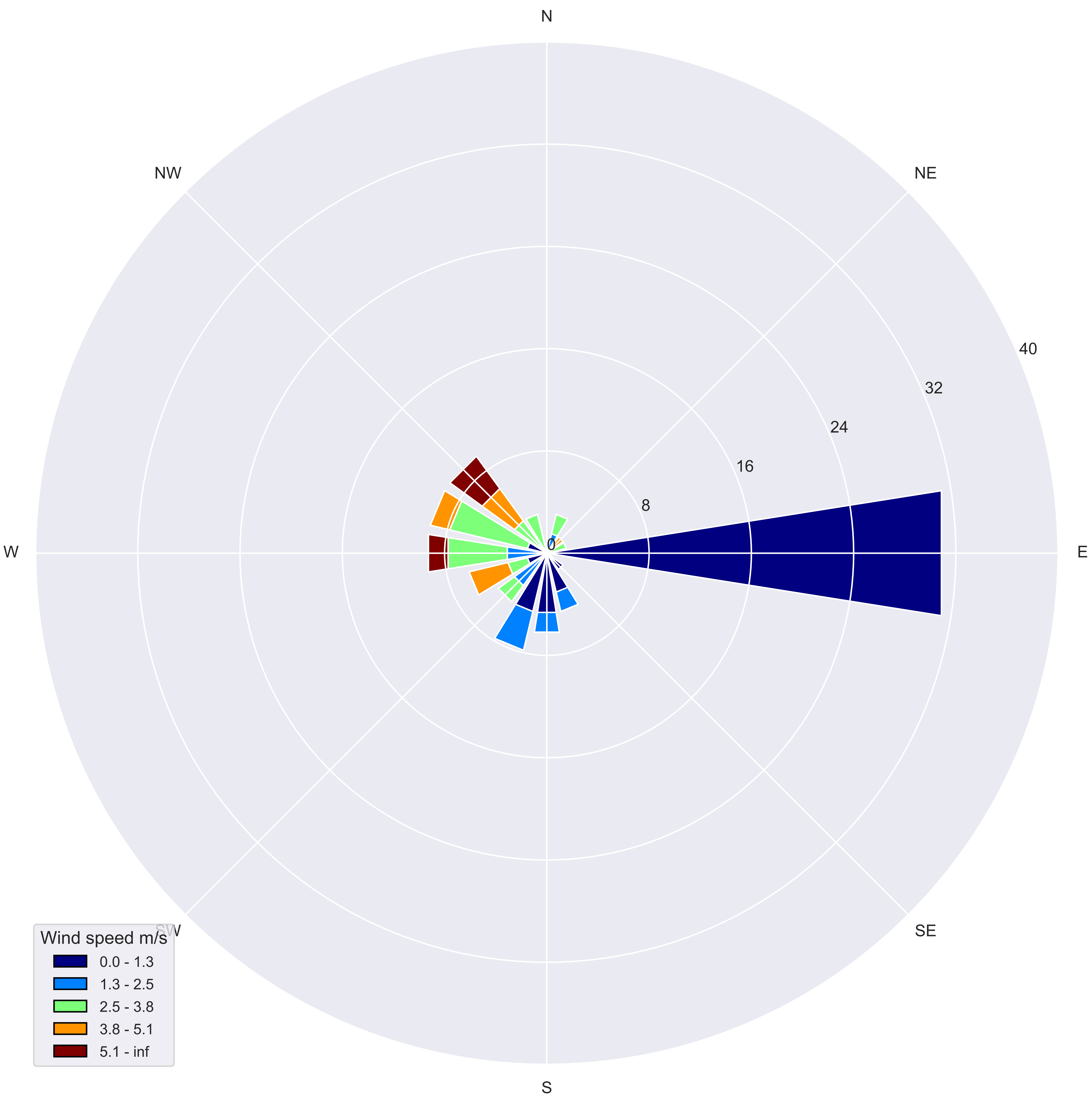

Supplement: S1 Fig — (PDF) [file pntd.0009357.s002.pdf]
