# Supplementary material for: Mark-release-recapture of male Aedes aegypti (Diptera: Culicidae): Use of rhodamine B to estimate movement, mating and population parameters in preparation for an incompatible male program
Source: PLoS Negl Trop Dis. 2021 Jun 7;15(6):e0009357. doi: 10.1371/journal.pntd.0009357 (PMC8183986; doi:10.1371/journal.pntd.0009357)

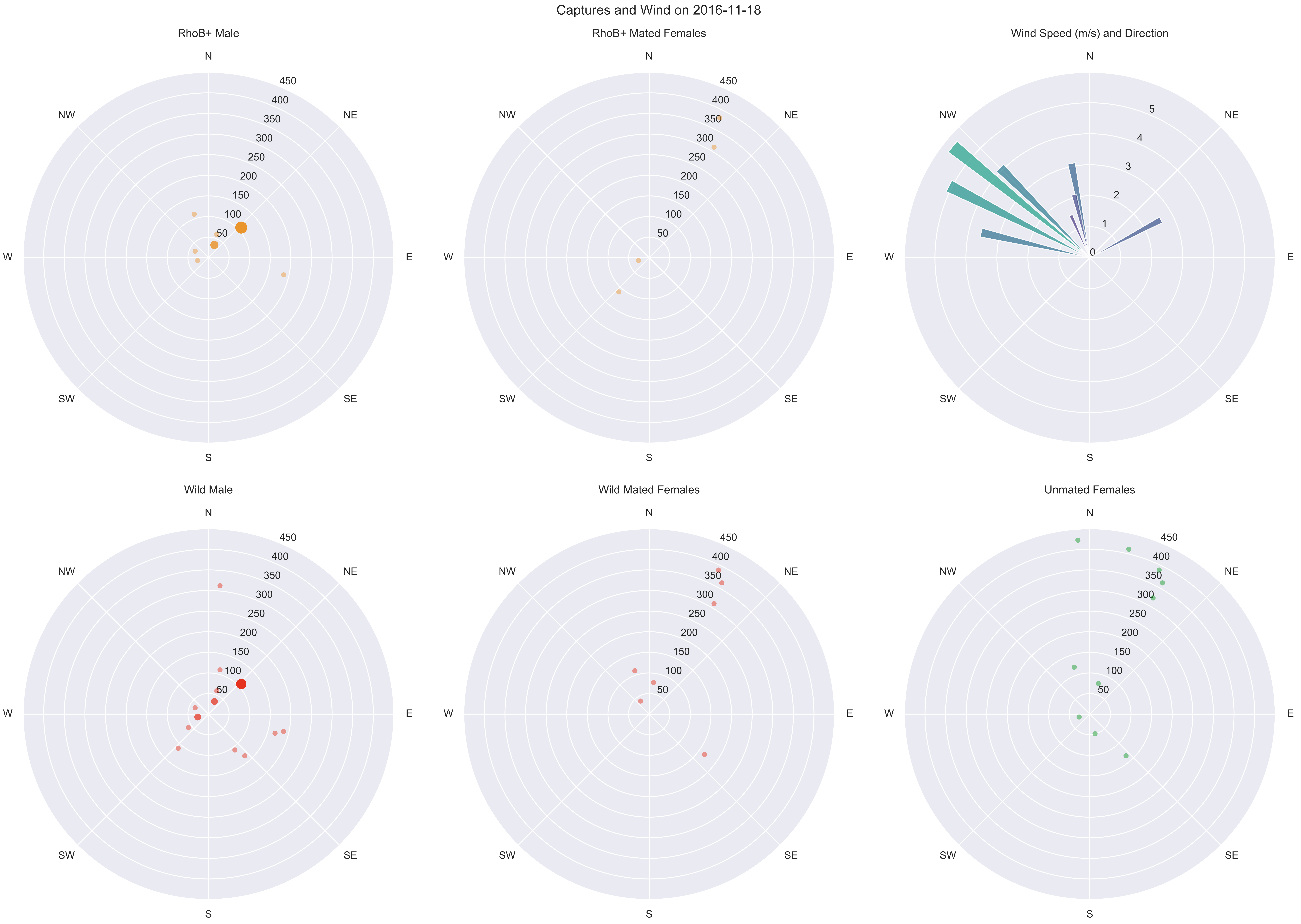

Captures and Wind on 2016-11-18
RhoB+ Male
RhoB+ Mated Females
Wind Speed (m/s) and Direction
Wild Male
Wild Mated Females
Unmated Females
N
NE
E
SE
S
SW
W
NW
50
100
150
200
250
300
350
400
450
0
1
2
3
4
5

Captures and Wind on 2016-11-19

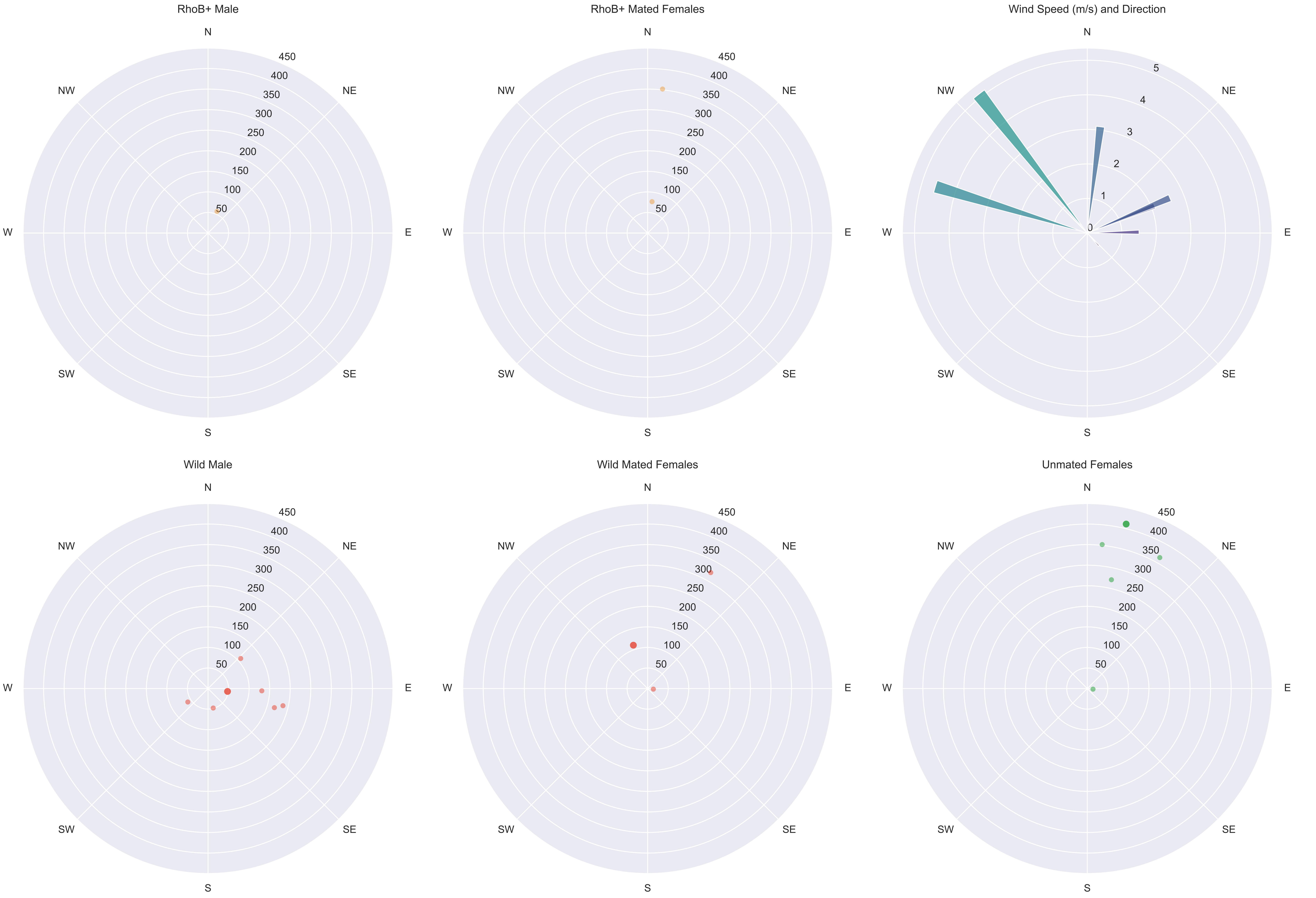

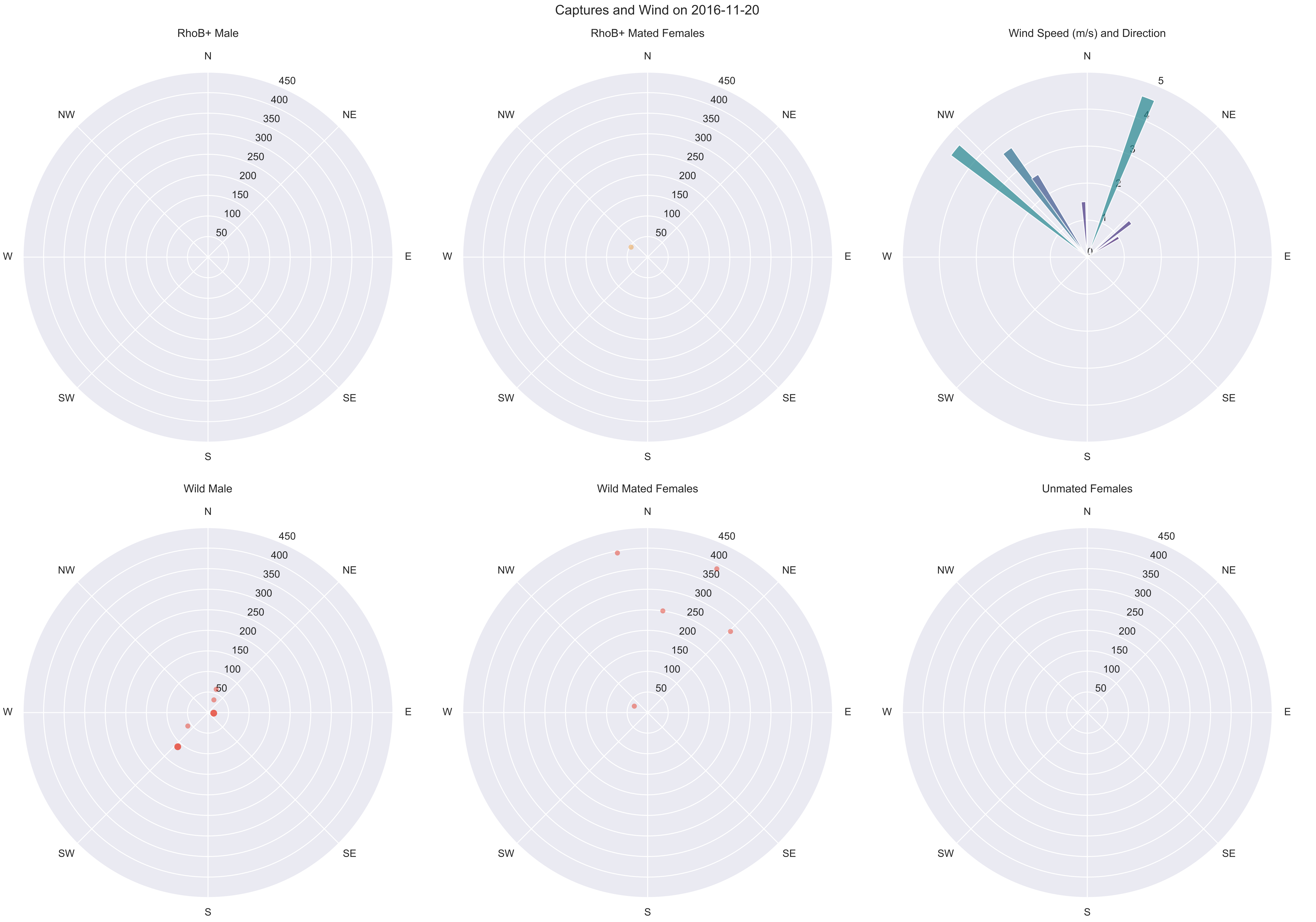
Captures and Wind on 2016-11-20
RhoB+ Male
RhoB+ Mated Females
Wind Speed (m/s) and Direction
Wild Male
Wild Mated Females
Unmated Females
N
NE
E
SE
S
SW
W
NW
50
100
150
200
250
300
350
400
450
0
1
2
3
4
5

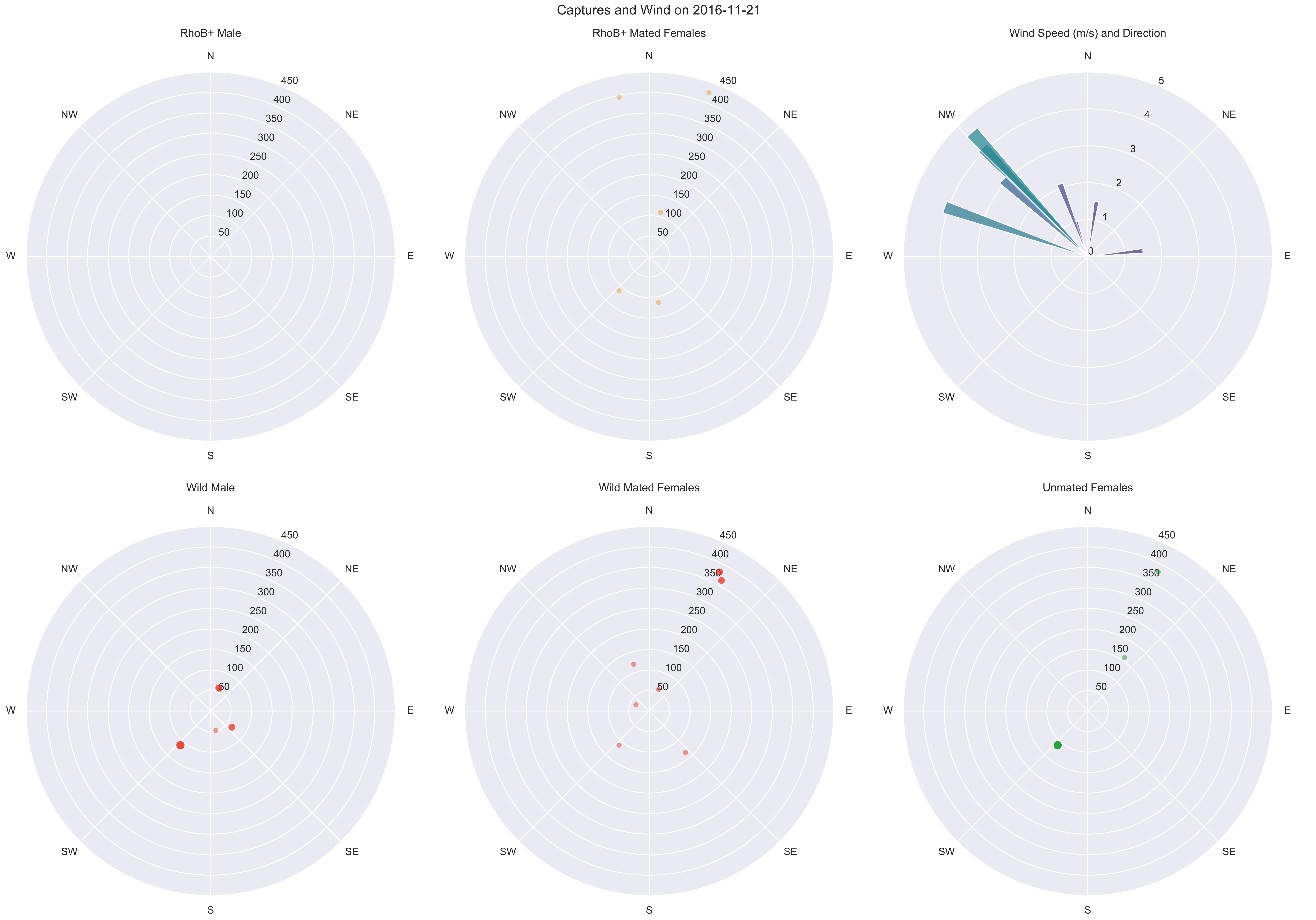

Captures and Wind on 2016-11-21
RhoB+ Male
RhoB+ Mated Females
Wind Speed (m/s) and Direction
Wild Male
Wild Mated Females
Unmated Females
N
NE
E
SE
S
SW
W
NW
50
100
150
200
250
300
350
400
450
0
1
2
3
4
5

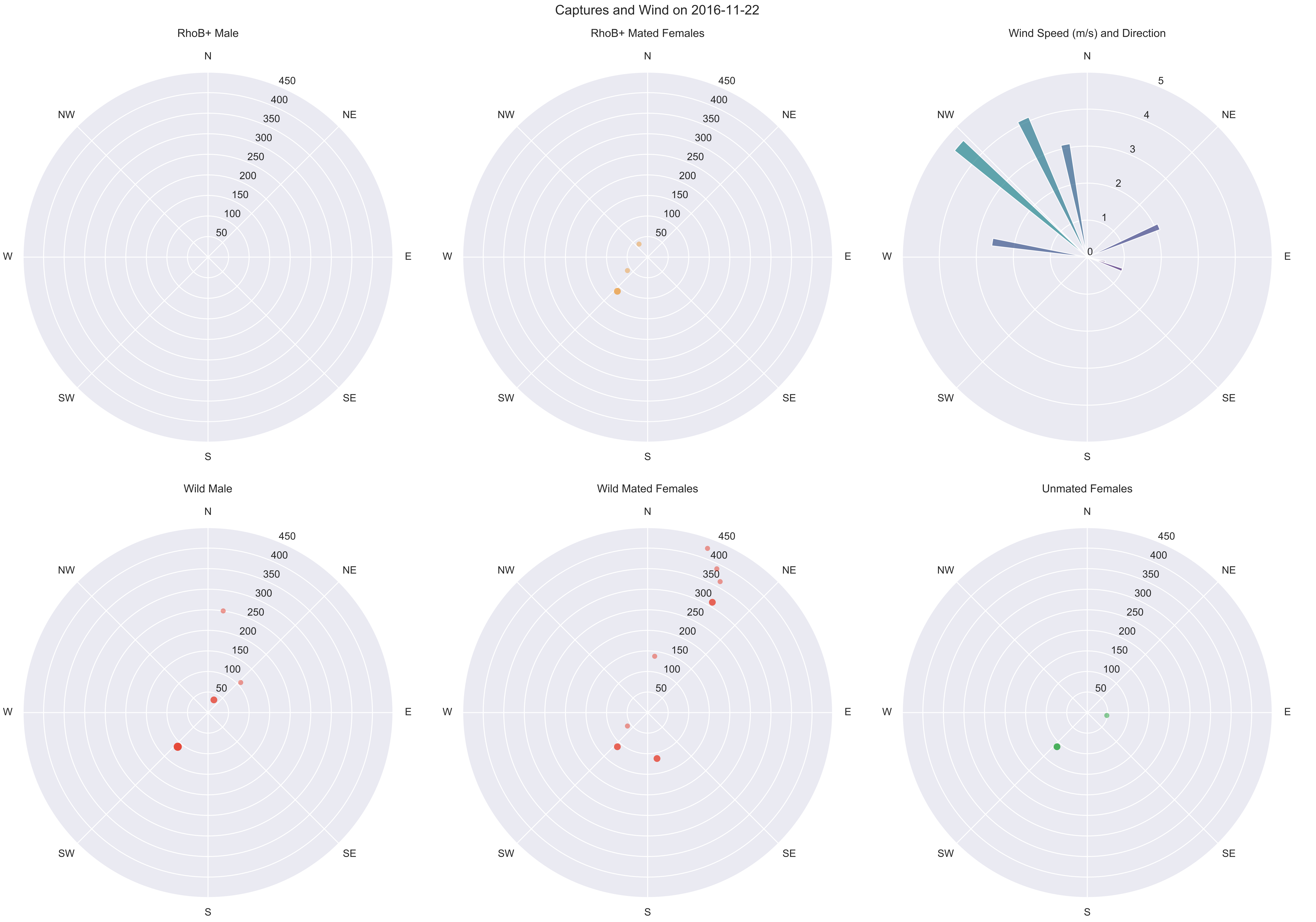
Captures and Wind on 2016-11-22
RhoB+ Male
RhoB+ Mated Females
Wind Speed (m/s) and Direction
Wild Male
Wild Mated Females
Unmated Females
N
NE
E
SE
S
SW
W
NW
50
100
150
200
250
300
350
400
450
0
1
2
3
4
5

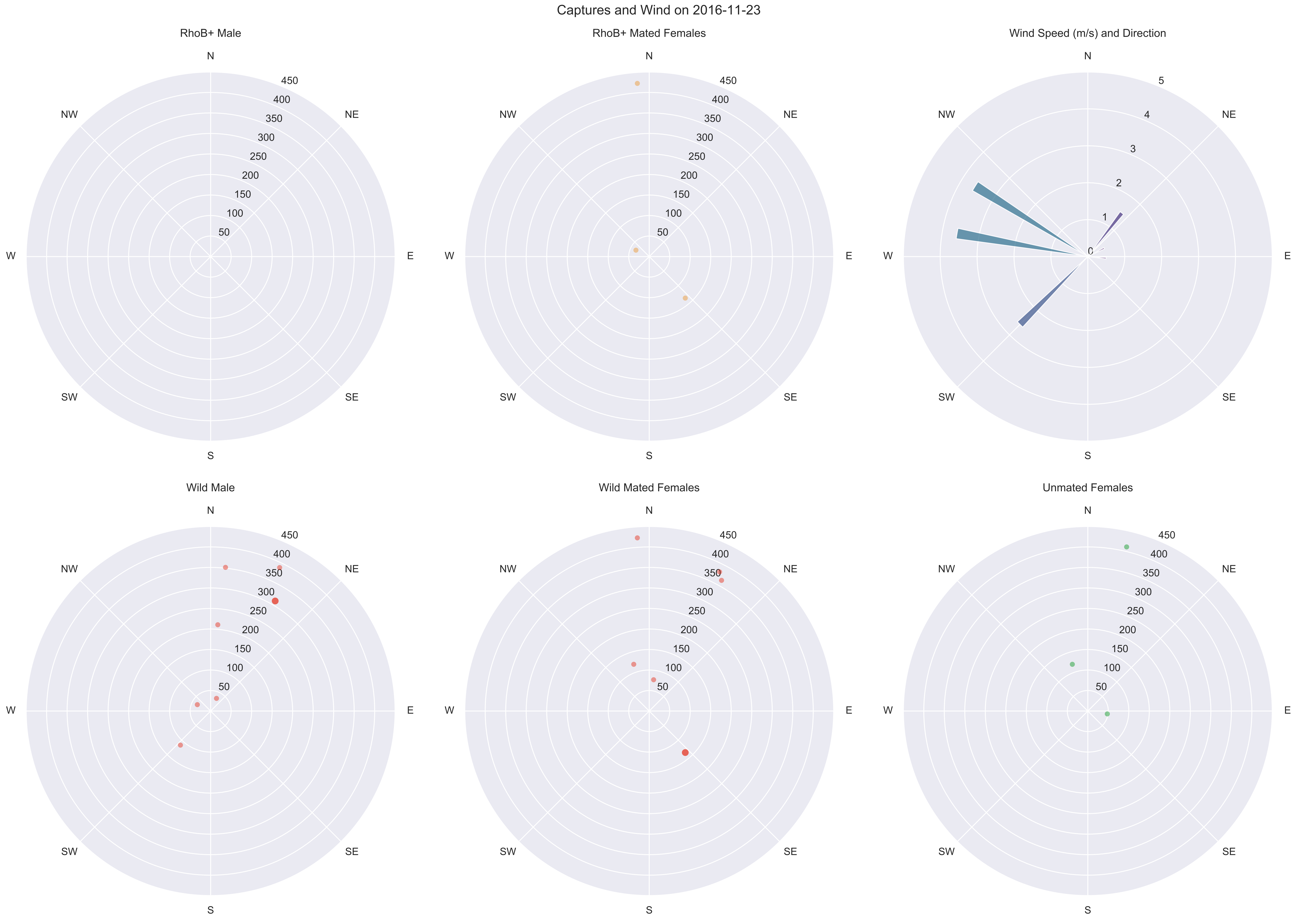
Captures and Wind on 2016-11-23
RhoB+ Male
RhoB+ Mated Females
Wind Speed (m/s) and Direction
Wild Male
Wild Mated Females
Unmated Females
N
NE
E
SE
S
SW
W
NW
50
100
150
200
250
300
350
400
450
0
1
2
3
4
5

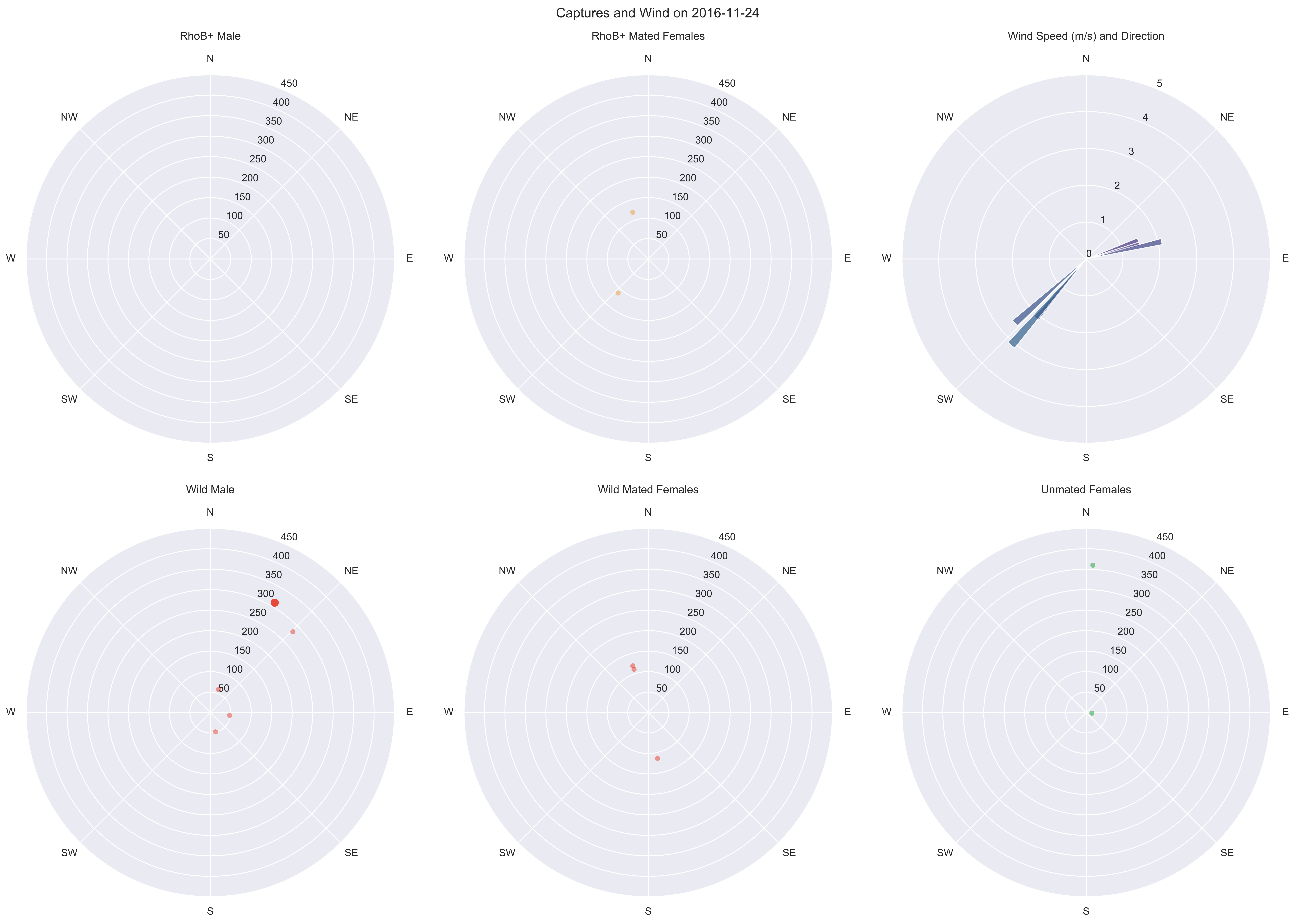
Captures and Wind on 2016-11-24
RhoB+ Male
RhoB+ Mated Females
Wind Speed (m/s) and Direction
Wild Male
Wild Mated Females
Unmated Females
N
NE
E
SE
S
SW
W
NW
50
100
150
200
250
300
350
400
450
0
1
2
3
4
5

Captures and Wind on 2016-11-27

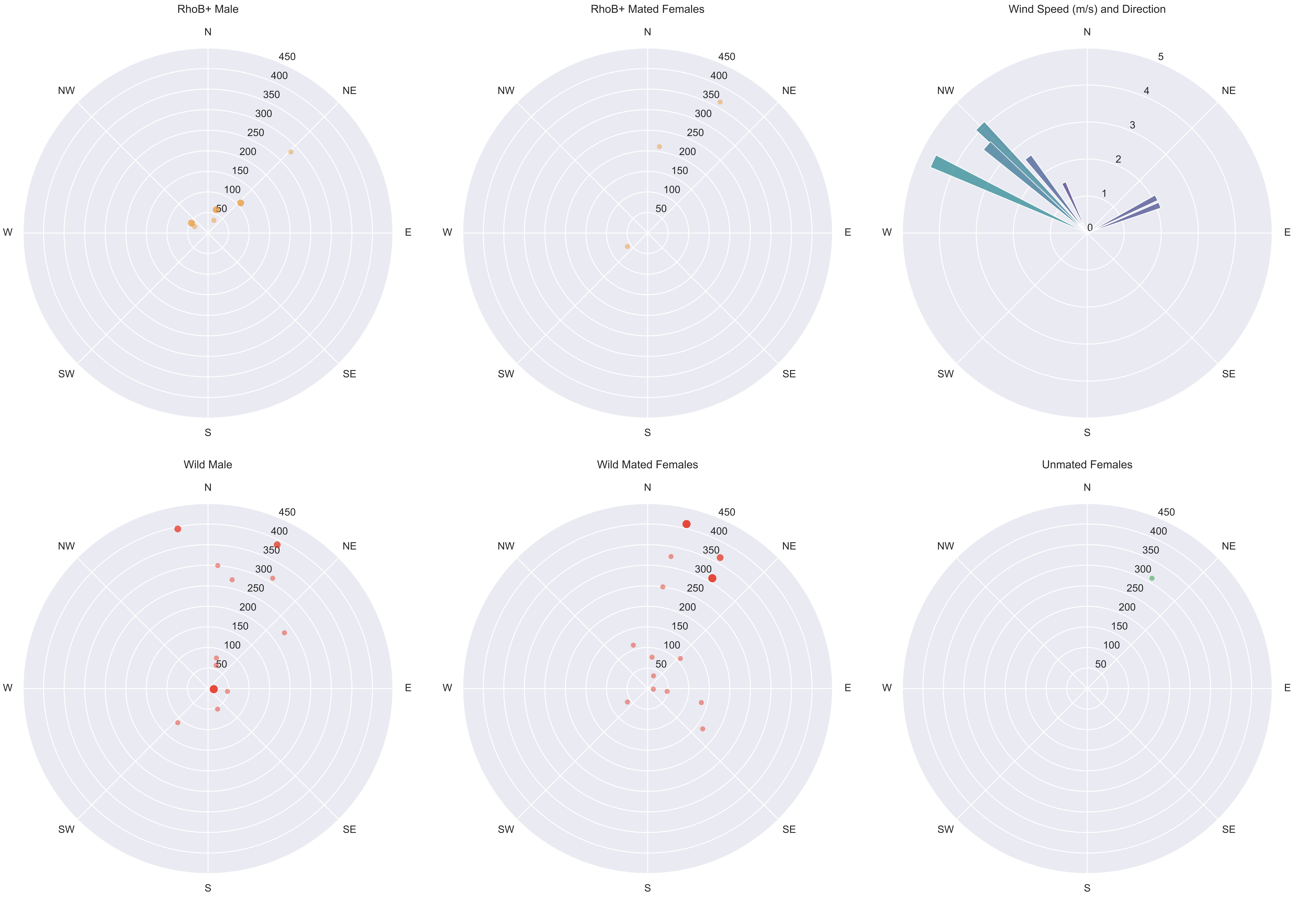

Captures and Wind on 2016-11-28

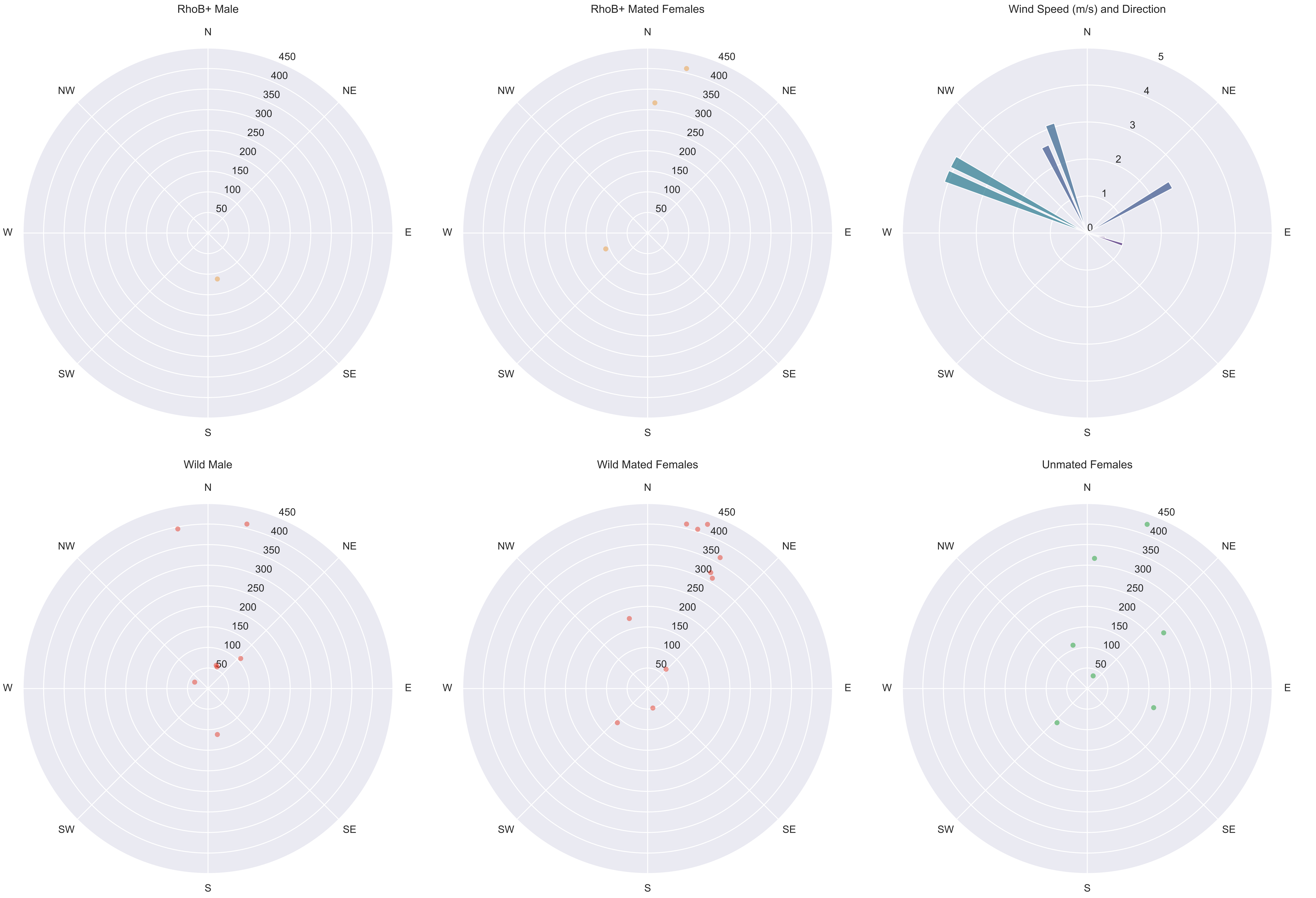

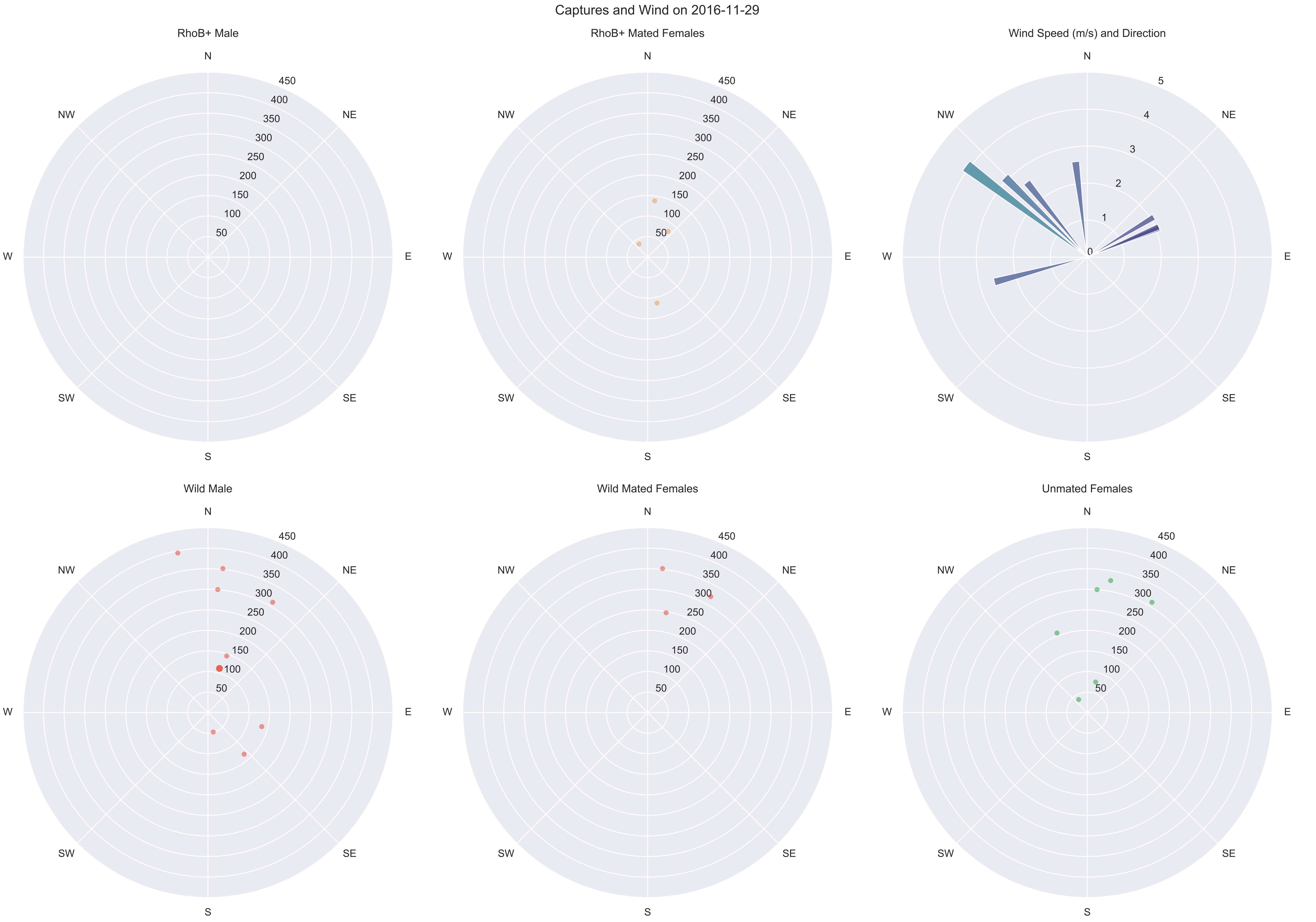

Captures and Wind on 2016-11-29
RhoB+ Male
RhoB+ Mated Females
Wind Speed (m/s) and Direction
Wild Male
Wild Mated Females
Unmated Females
N
NE
E
SE
S
SW
W
NW
50
100
150
200
250
300
350
400
450
0
1
2
3
4
5

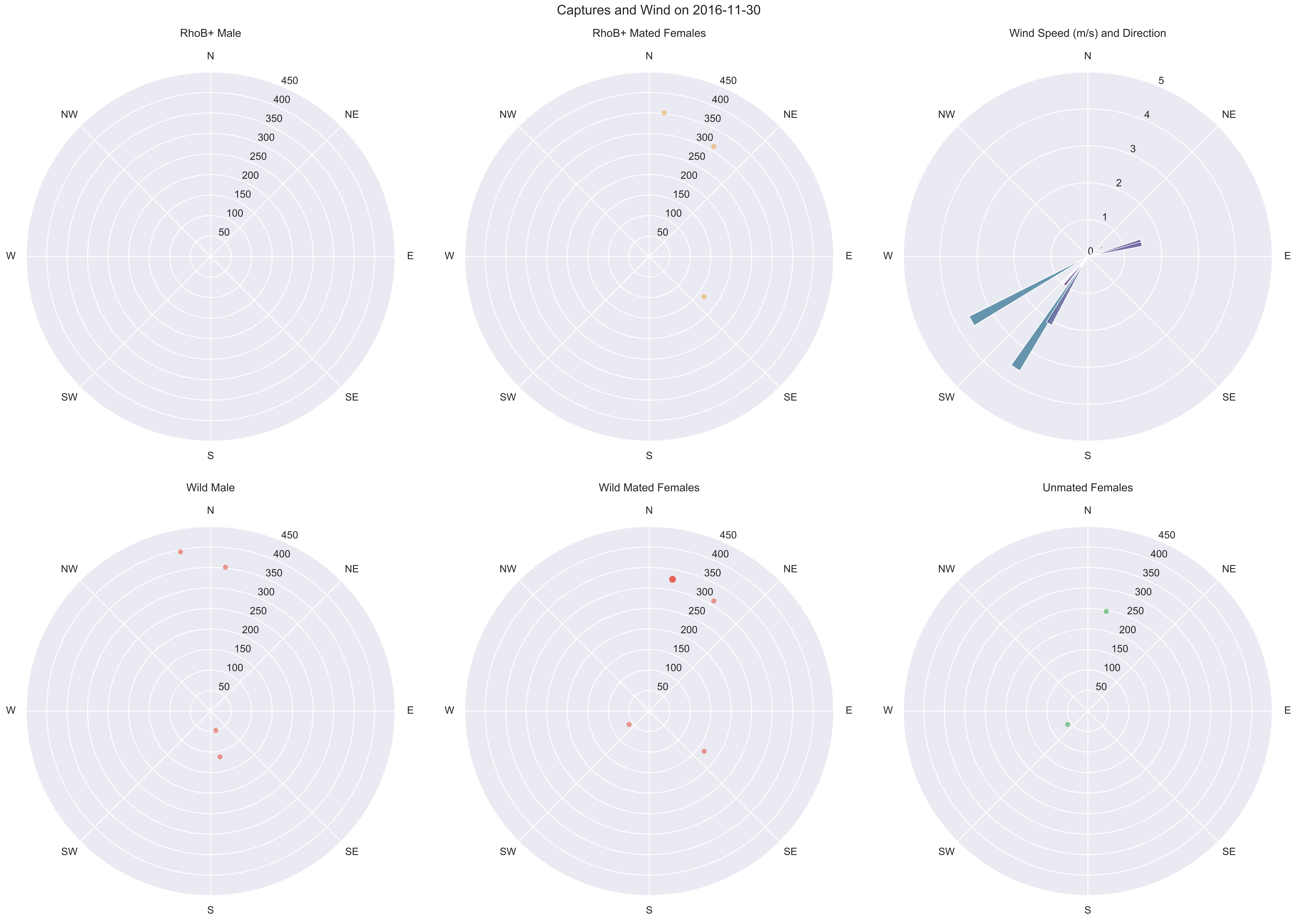
Captures and Wind on 2016-11-30
RhoB+ Male
RhoB+ Mated Females
Wind Speed (m/s) and Direction
Wild Male
Wild Mated Females
Unmated Females
N
NE
E
SE
S
SW
W
NW
50
100
150
200
250
300
350
400
450
0
1
2
3
4
5

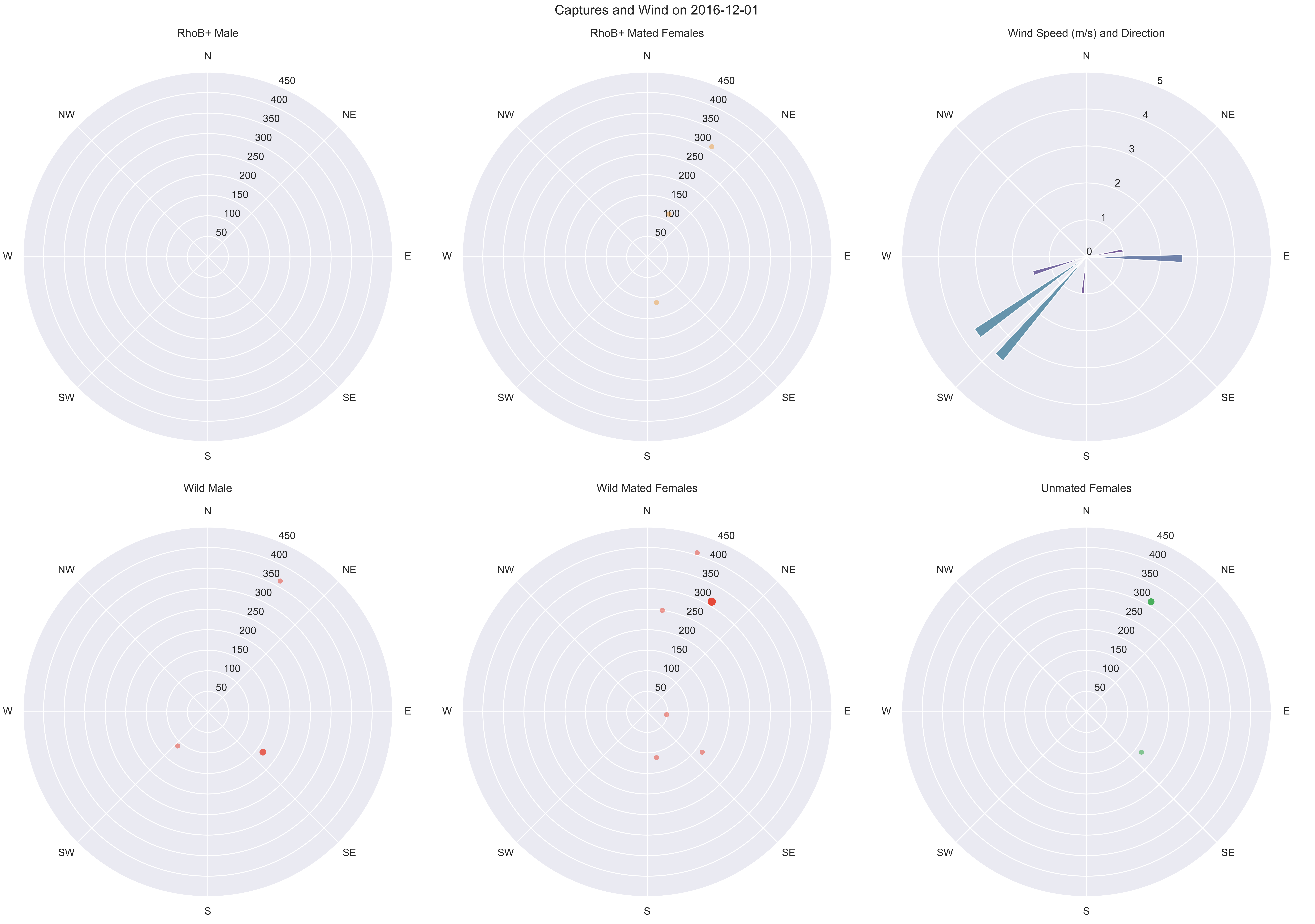
Captures and Wind on 2016-12-01
RhoB+ Male
RhoB+ Mated Females
Wind Speed (m/s) and Direction
Wild Male
Wild Mated Females
Unmated Females
N
NE
E
SE
S
SW
W
NW
50
100
150
200
250
300
350
400
450
0
1
2
3
4
5

Captures and Wind on 2016-12-02

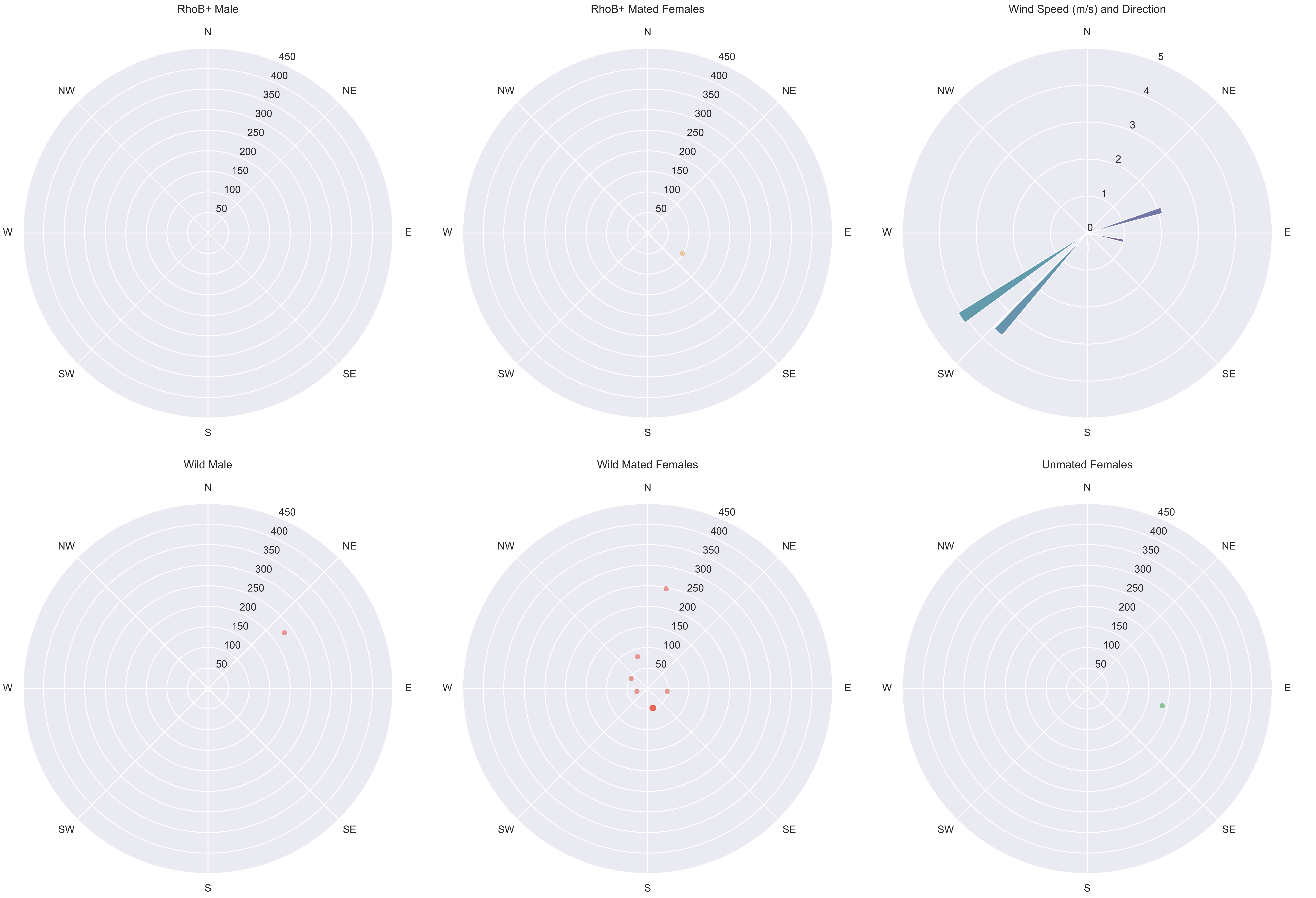

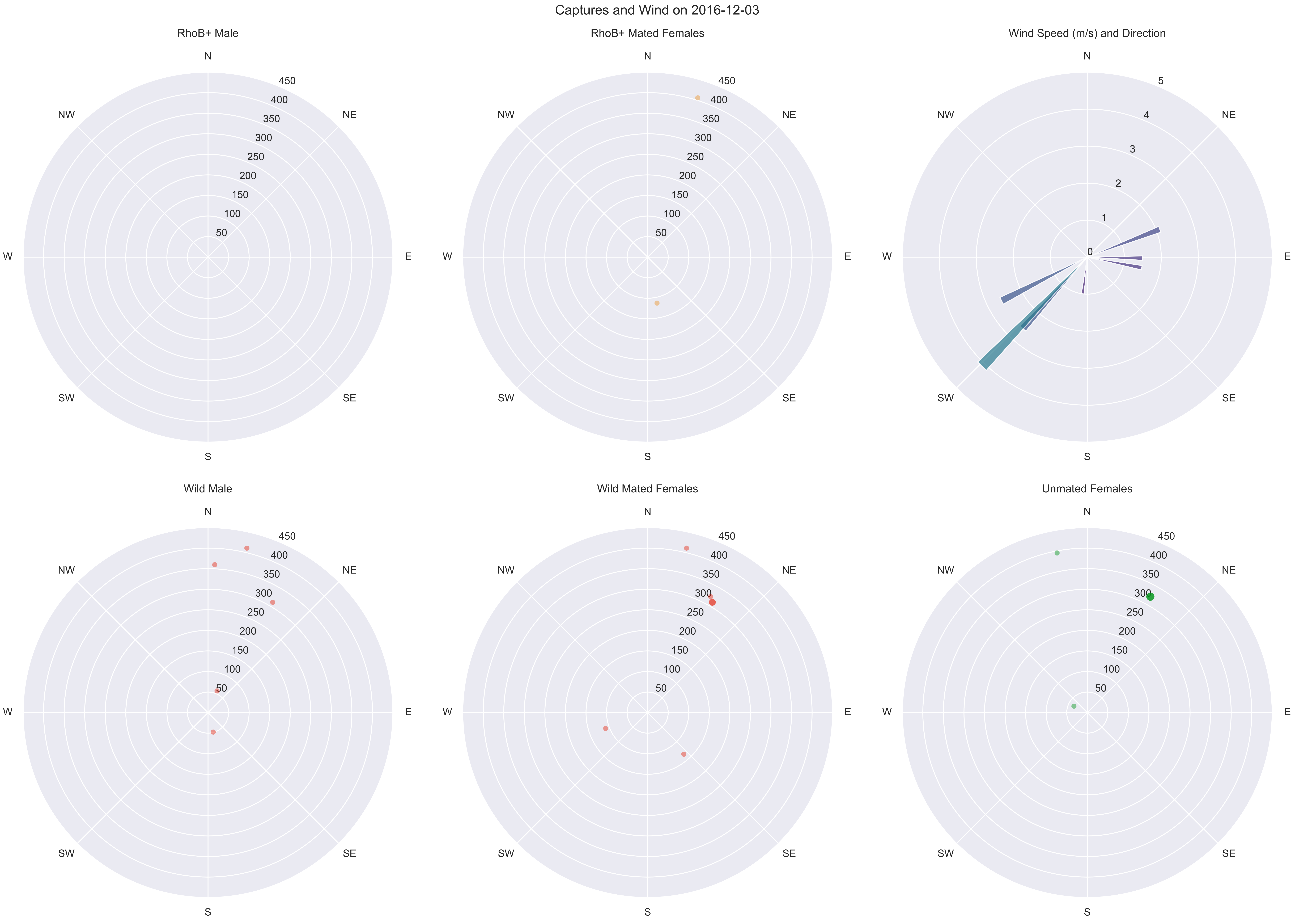
Captures and Wind on 2016-12-03
RhoB+ Male
RhoB+ Mated Females
Wind Speed (m/s) and Direction
Wild Male
Wild Mated Females
Unmated Females
N
NE
E
SE
S
SW
W
NW
50
100
150
200
250
300
350
400
450
0
1
2
3
4
5

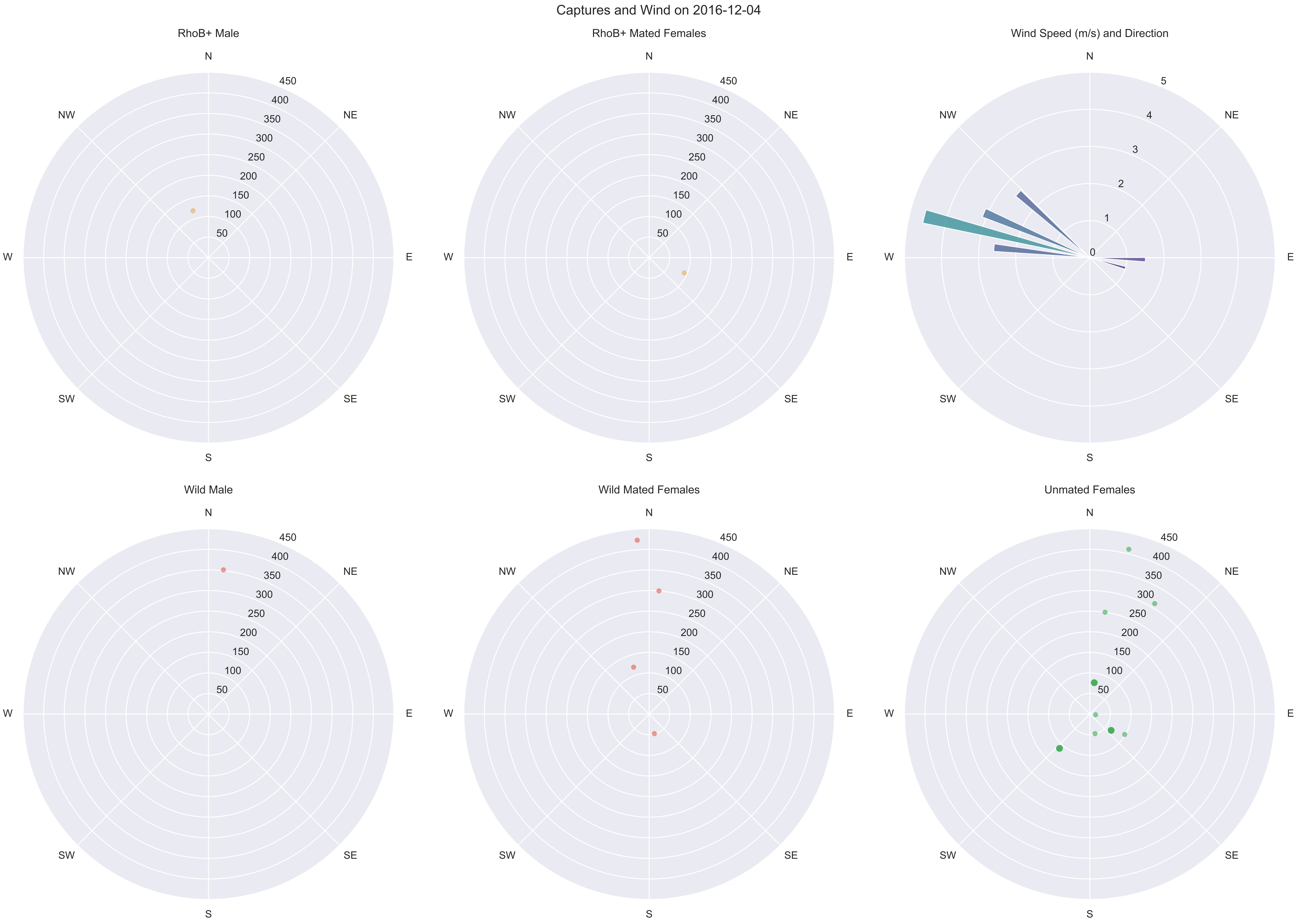
Captures and Wind on 2016-12-04
RhoB+ Male
RhoB+ Mated Females
Wind Speed (m/s) and Direction
Wild Male
Wild Mated Females
Unmated Females
N
NE
E
SE
S
SW
W
NW
50
100
150
200
250
300
350
400
450
0
1
2
3
4
5

Captures and Wind on 2017-02-22

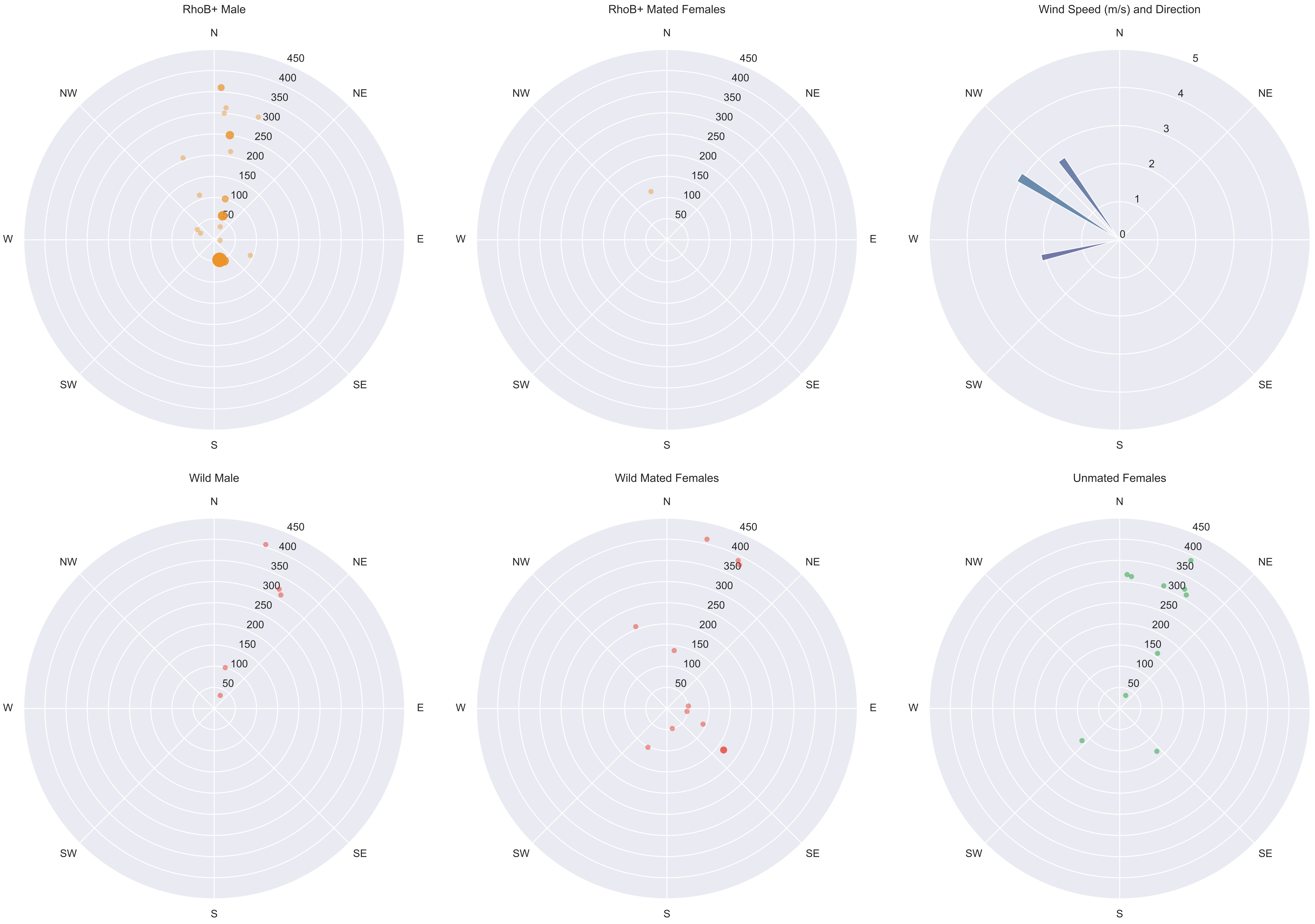

Captures and Wind on 2017-02-23

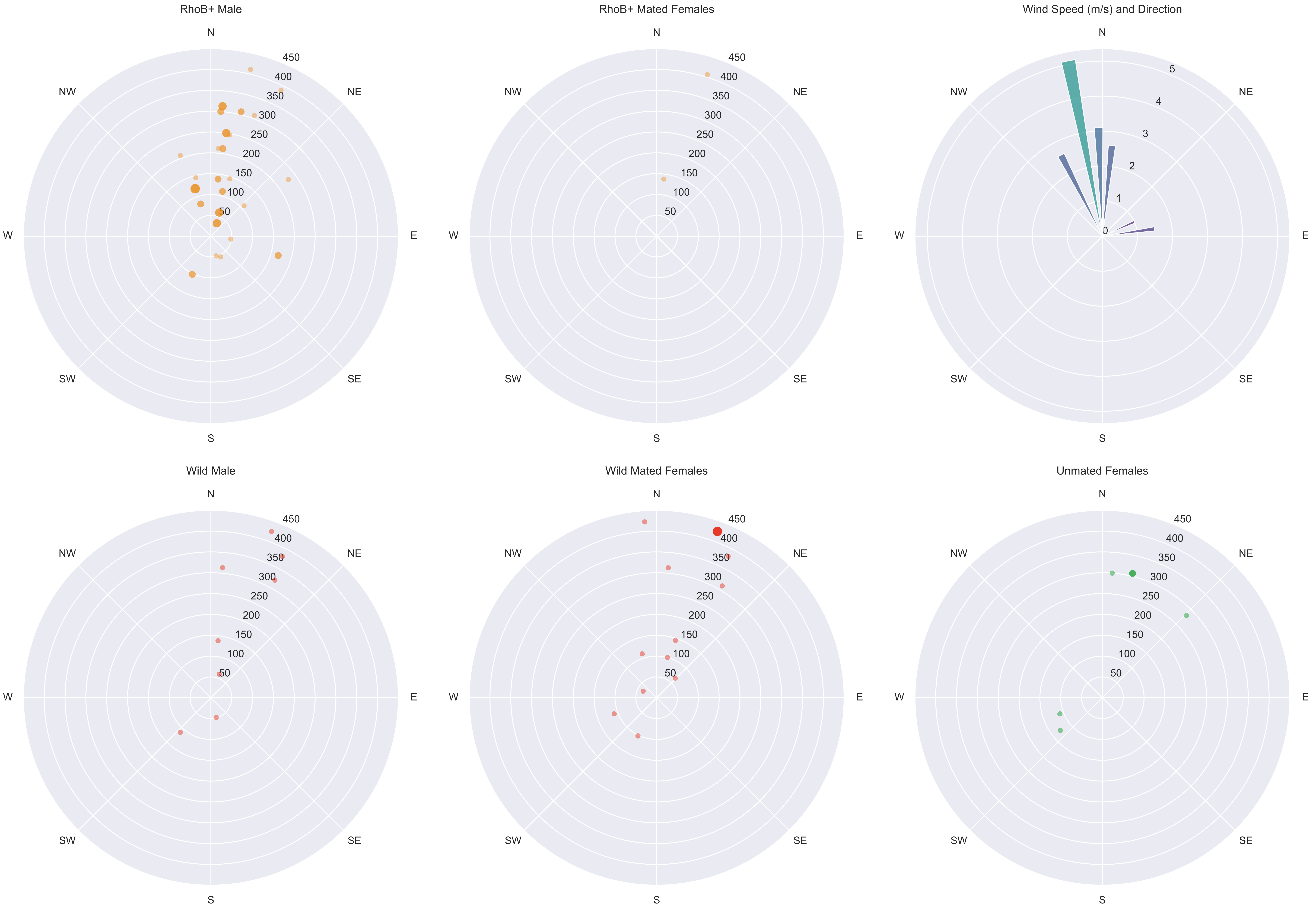

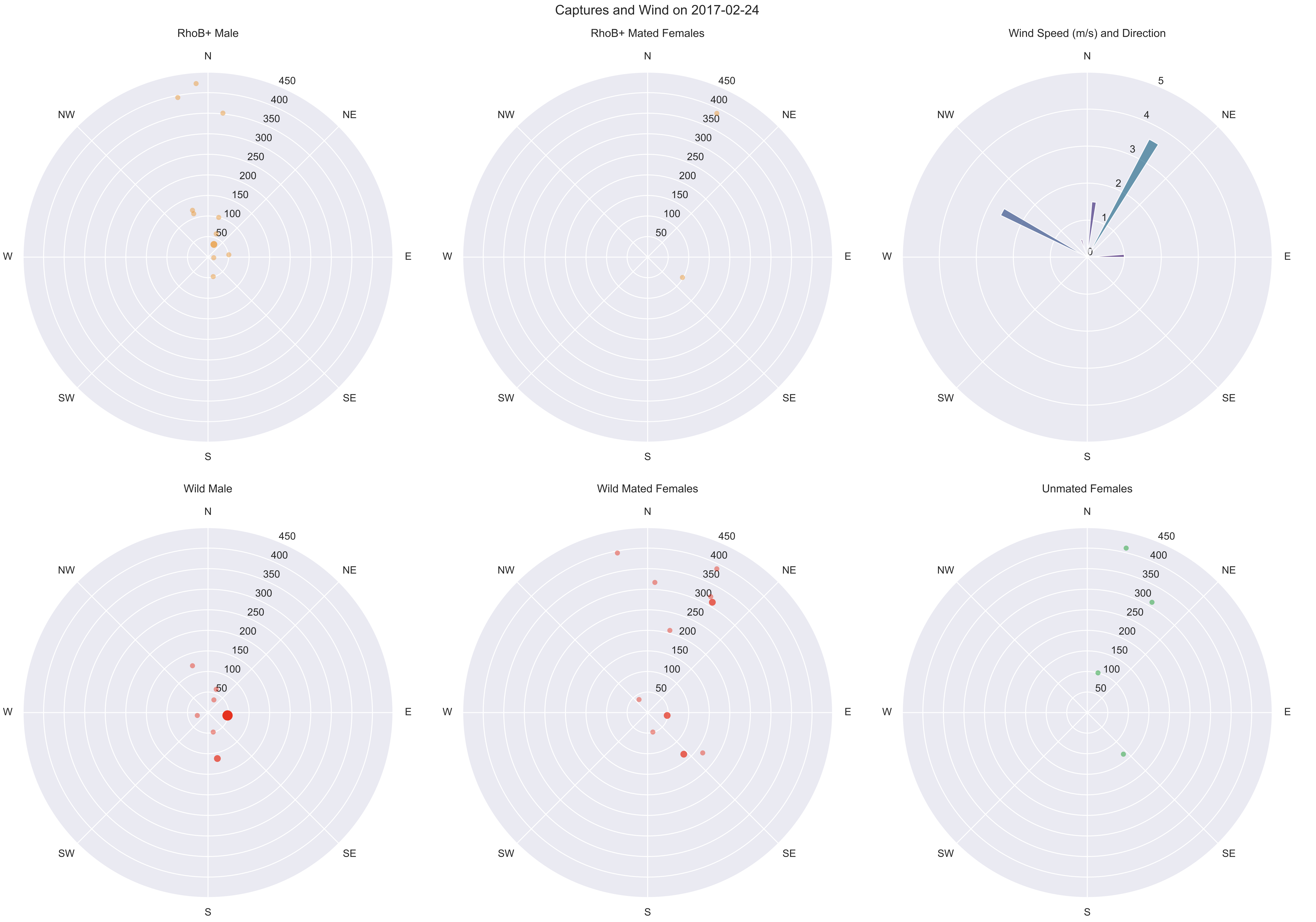
Captures and Wind on 2017-02-24
RhoB+ Male
RhoB+ Mated Females
Wind Speed (m/s) and Direction
Wild Male
Wild Mated Females
Unmated Females
N
NE
E
SE
S
SW
W
NW
50
100
150
200
250
300
350
400
450
0
1
2
3
4
5

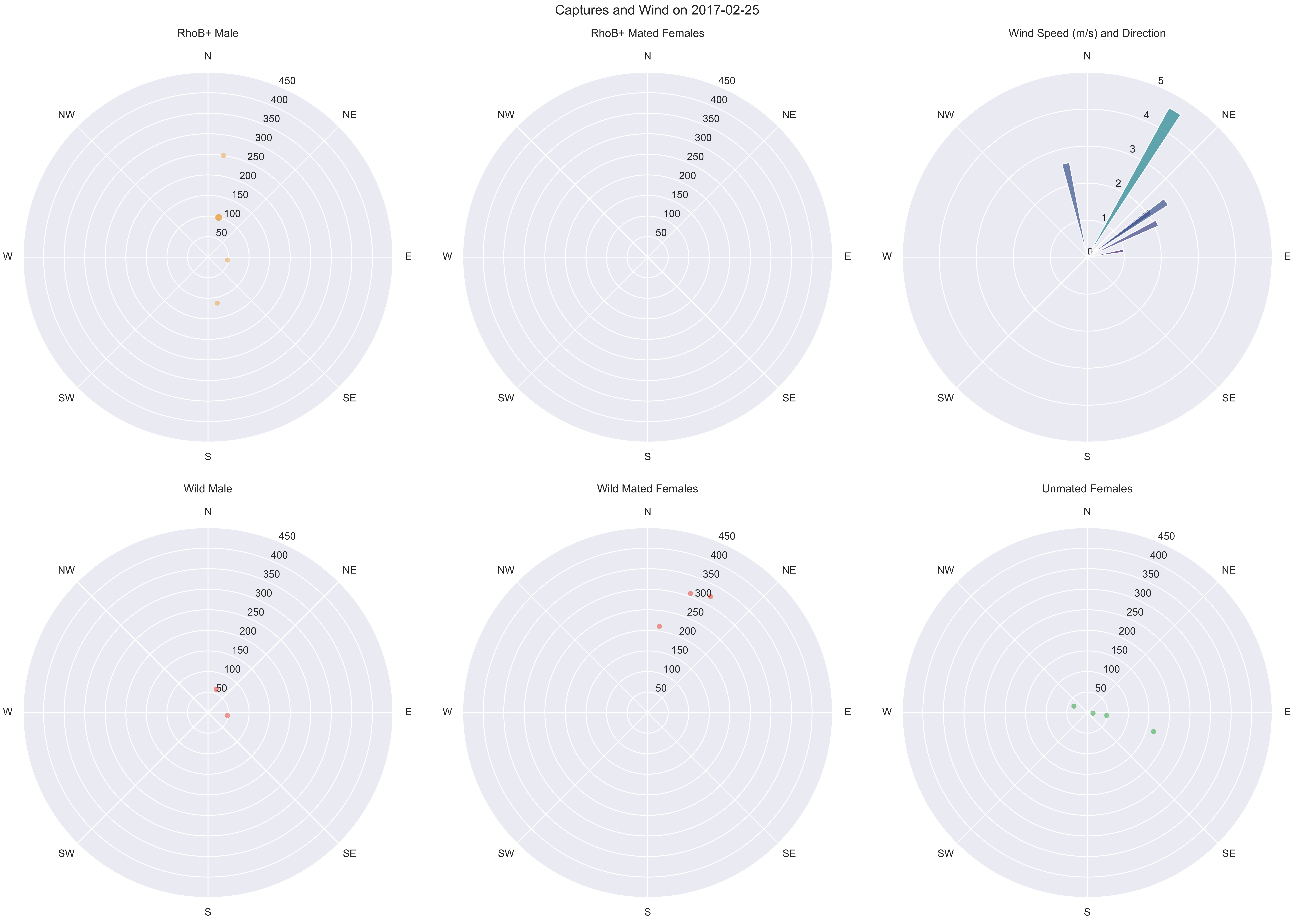
Captures and Wind on 2017-02-25
RhoB+ Male
RhoB+ Mated Females
Wind Speed (m/s) and Direction
Wild Male
Wild Mated Females
Unmated Females
N
NE
E
SE
S
SW
W
NW
50
100
150
200
250
300
350
400
450
0
1
2
3
4
5

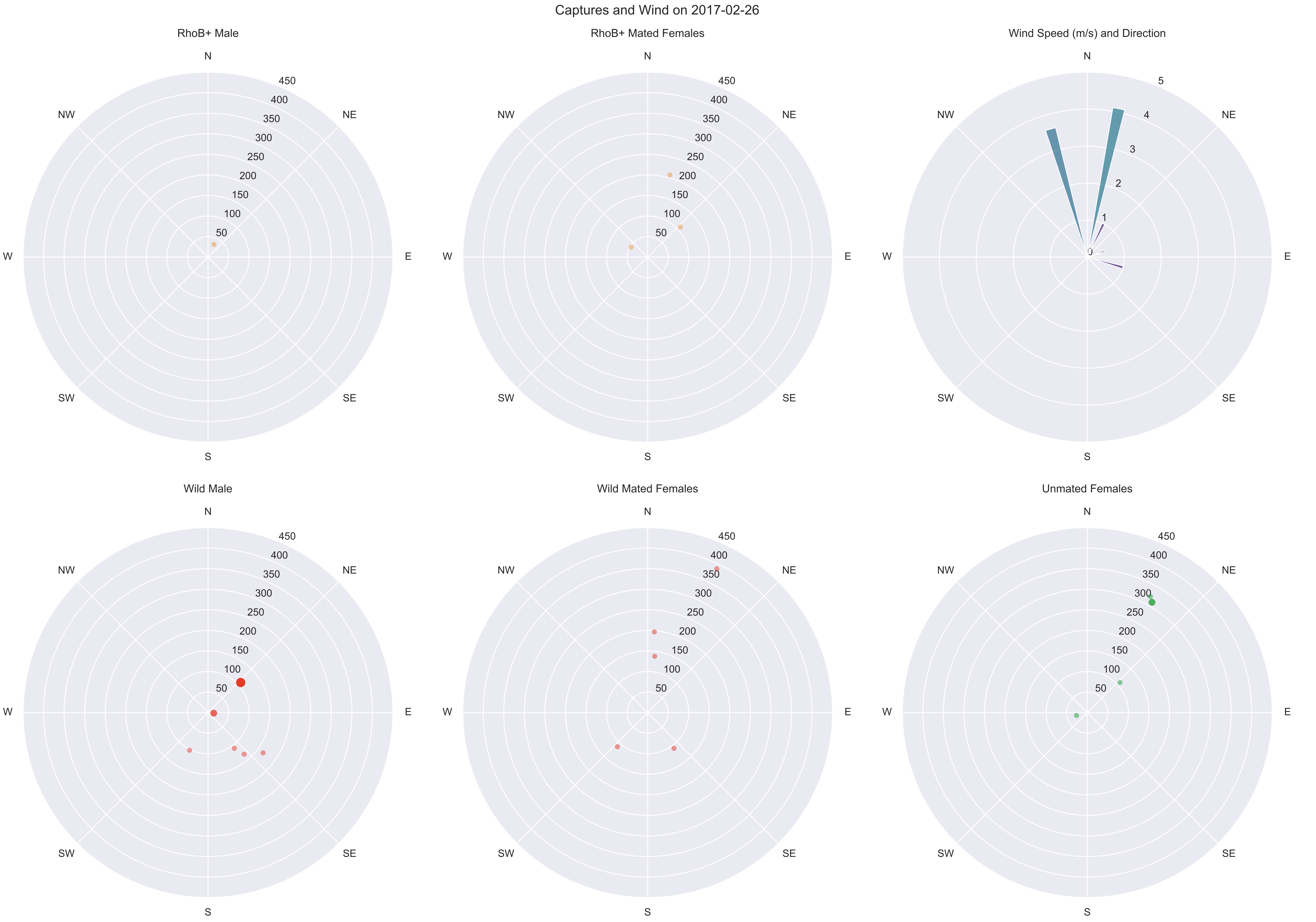

Captures and Wind on 2017-02-26
RhoB+ Male
RhoB+ Mated Females
Wind Speed (m/s) and Direction
Wild Male
Wild Mated Females
Unmated Females
N
NE
E
SE
S
SW
W
NW
50
100
150
200
250
300
350
400
450
0
1
2
3
4
5

Captures and Wind on 2017-02-27

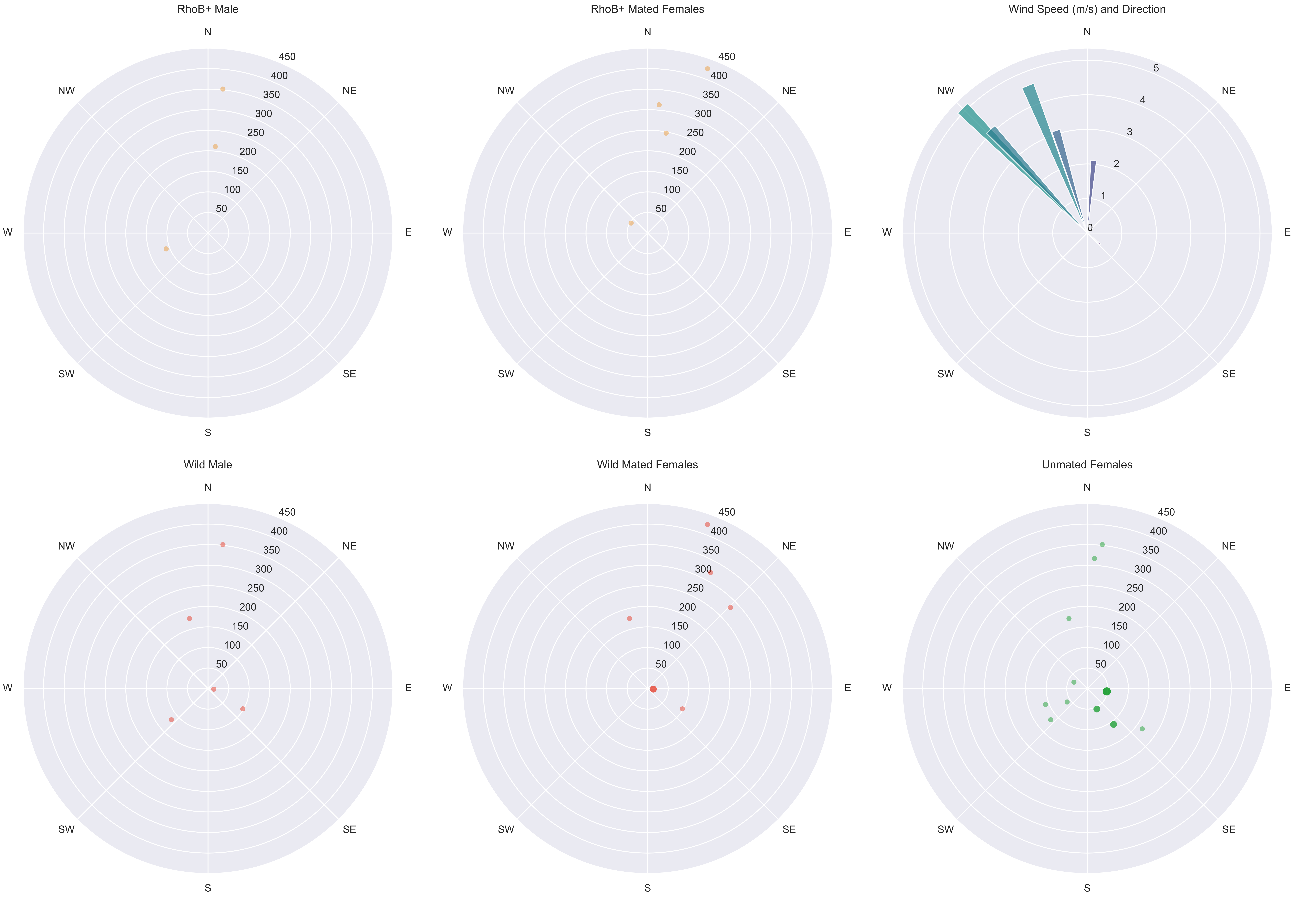

Supplement: S2 Fig — (PDF) [file pntd.0009357.s003.pdf]
