# Supplementary material for: Mark-release-recapture of male Aedes aegypti (Diptera: Culicidae): Use of rhodamine B to estimate movement, mating and population parameters in preparation for an incompatible male program
Source: PLoS Negl Trop Dis. 2021 Jun 7;15(6):e0009357. doi: 10.1371/journal.pntd.0009357 (PMC8183986; doi:10.1371/journal.pntd.0009357)
Supplement: Attachment — Submitted filename: Reisen et al. 1981. An. culicifacies mating and comp.Ann ESA 74_395.pdf [file pntd.0009357.s004.pdf]

Anopheles culicifacies Giles:[1] Mating Behavior and Competitiveness in Nature of Chemosterilized Males Carrying a Genetic Sexing System[2]

WILLIAM K. REISEN[3], RICHARD H. BAKER[4], RICHARD K. SAKAI, FARIDA MAHMOOD, HAMAYUN R. RATHOR, KOUKAB RAANA, AND GHAZALA TOQIR

Pakistan Medical Research Center, 6, Birdwood Road, Lahore-3, Pakistan, and International Health Program, University of Maryland School of Medicine, Baltimore, Maryland 21201

ABSTRACT

Ann. Entomol. Soc. Am. 74: 395–401 (1981)

Chemosterilized *Anopheles culicifacies* Giles males carrying a Y-linked, dieldrin genetic sexing device (D-13) were released concomitantly with a laboratory-adapted strain (SAT) and the progeny of wild-caught females (KB) as pupae through a self-dusting device positioned at a breeding site near Kot Baghicha Singh Walla, Pakistan during April 1980. Some dusted SAT and KB ♀♀ were also released as virgin adults inside diurnal cattle shed resting sites. Emerging D-13 ♂♂ behaved similarly to endemic unmarked males (UM); i.e., they rested outdoors until sexually mature, immigrated into a cattle-shed compound where they swarmed with UM ♂♂ and rested indoors on the following day in equal proportions to swarm composition. In addition, equal proportions of D-13 and UM ♂♂ mated at each collection site. However, estimates of mating competitiveness based on the fertility of egg batches from individual SAT, KB and UM ♀♀ were statistically less than one, suggesting that D-13 ♂♂ were less competitive in nature, although equally competitive under laboratory conditions.

With the advent of insecticide resistence in *Anopheles culicifacies* Giles (e.g., Manoucheri et al. 1975, Rajagopal 1977) accompanying the resurgence of human malaria in south Asia (e.g., Akhtar et al. 1977), recent research has been directed at developing alternative methods of control including genetics. In Pakistan, Baker et al. (1980) have demonstrated that *A. culicifacies* males carrying a complex chromosomal rearrangement in a laboratory strain background were equally competitive for concomitantly released laboratory strain females and the unselected progeny of wild-caught females; i.e., assortative mating had not evolved due to colonization or maintenance or both. However, few of the aberration-carrying males released as adults inside diurnal, cattle shed resting sites mated with unmarked, endemic females, suggesting that an adequate number of males was not released at the correct time and place. In addition, the release of complex male-linked translocations for population suppression seemed impractical due to: (1) the greater production effort caused by the high male-linked sterility, (2) the necessity of laborious manual sexing to remove fertile and potentially dangerous females, and (3) the degree of sterility imparted after mating with wild females. Perhaps a more efficient approach would be the incorporation of a genetic sexing system to produce only males which could be sterilized before release (Bailey et al. 1980). Such a system has recently been developed for *A. culicifacies* by translocating the gene for dieldrin resistance (chromosome 3) to the Y-chromosome (Baker et al. 1981). By back-crossing these males to homozygous dieldrin-susceptible females, males may be selectively produced by treating first-stage larvae for 24 h with dieldrin at 0.02 ppm. The emphasis of the present experiment was to study the behavior and mating competitiveness in nature of male *A. culicifacies* arising from pupae which had been sexed by the genetic system and then treated with the chemosterilant bisazir.

Materials and Methods

Description of Study Area and Test Population

Mosquitoes were released during April 1980 near the village of Kot Baghicha Singh Walla, Punjab Province, Pakistan, at the same cattle shed-feedlot compound used in previous experiments (Baker et al. 1980). Pupae were released along the margin of an irrigation-well water-course breeding site having a relative abundance of 4.4 *A. culicifacies* larvae per dip. Adult females were released inside cattle sheds having a resting relative abundance of 88.4 ♀♀ and 58.4 ♂♂ per man-hour of collection effort. Temperature and relative humidity measured inside the cattle sheds during afternoon averaged 27°C and 79% whereas temperature and relative humidity after sunset were 23°C and 78%, respectively. On 2 April 1980, a wind/dust/rain storm occurred depositing 13 mm rain.

Strains.—The following strains were used throughout. (1) D-13: Genetic sexing strain produced by translocating the gene for dieldrin resistance on chromosome 3 to the Y-chromosome and maintained by backcrossing to homozygous dieldrin susceptible females (Baker et al. 1981). (2) SAT: Sattoki, Punjab Province, laboratory-adapted strain colonized by Ainsley (1976). (3) KB: Progeny of wild-caught females from Kot Baghicha Singh Walla which were reared in the laboratory. (4) UM: Unmarked adults collected at Kot Baghicha.

Production.—First-stage D-13 larvae were exposed to dieldrin at 0.02 ppm for 24 h after eclosion. The surviving ♂ larvae were then diluted into a series of covered enamel pans in which they were fed a daily ration of finely sifted liver powder. Larval-pupal separation

[1] Diptera: Culicidae.

[2] Received for publication November 21, 1980.

[3] Present address: Arbovirus Field Station, Univ. of Calif. School of Public Health, Box 1564, Bakersfield, CA, 93302.

[4] Please address reprint requests to Dr. Richard H. Baker, International Health Program, Univ. of Md. School of Medicine, 10 South Pine Street, Baltimore, MD, 21201.

©1981 Entomological Society of America

0013-8746/81/0403-9507$02.00/0

was done manually or using an ice-water technique (Weathersby 1963). Pupae were then treated with a 1% solution of bisazir (Ent-61585) for 1 h and rinsed with well-water, and the numbers produced were estimated volumetrically (mean = 306.5 ± 5.69 pupae per ml) (Mukhtar et al. 1980).

Progeny of SAT and KB ♀♀ were reared in pans and fed liver powder. Some pupae were sexed and counted, whereas others were allowed to emerge. Adults were anesthetized with carbon dioxide, sexed and counted manually, marked with a cohort-specific fluorescent dust (Yasuno et al. 1972), and then held for 1 or 4 days in cages with a 3% sucrose source. All mosquitoes were reared and held under the natural photoperiod.

Release.—Chemosterilized D-13 ♂♂ and some SAT and KB ♀♀ were released as pupae in a self-marking device (Singh and Yasuno 1971) positioned along a water-course breeding site to simulate the natural situation (see Fig. 1 of Baker et al. 1980). Each female cohort was marked a specific color. All D-13 ♂♂ were marked pink, with the exception of those released on 1 April, which were marked blue. At Kot Baghicha, cool night temperatures (mean minimum = 18.9°C) delayed maturation and 2 days were required for the emergence of each cohort. Mosquitoes failing to emerge, disperse or both from the self-marking device were subtracted from the number measured volumetrically to estimate the number of adults emerging into the study area.

Marked adult females were released inside diurnal cattle shed resting sites at shed C compound (see Fig. 1 Baker et al. 1980) after indoor resting collections were completed, but at least 1 h before sunset (the time of crepuscular egress; Reisen et al. 1976). Specimens failing to disperse from the release cartons were subtracted from the numbers dusted to estimate the number released.

Recapture and Assessment.—Starting on the afternoon after the day of release, mosquitoes were recaptured by using the following three methods. (1) Adult mosquitoes resting in sheds C-1 to C-5 and D (Fig. 1 of Baker et al. 1980) were collected each afternoon with mouth aspirators (two collectors for 15 min per shed) and a mechanical sweeper (one collector for 10 min per shed) similar to that described by Davis and Gould (1973). (2) All mosquitoes resting in a Muirhead-Thompson (1958) pit shelter located between the pupal release site and shed C compound were collected each afternoon. (3) Starting about 15 min before sunset, one to three collectors sampled swarms of male *A. culicifacies* which formed in the vicinity of indoor resting sites by using an aerial net. Collections were kept separate by swarm.

All collections were held at 5°C overnight and then etherized, examined for fluorescent dust under ultraviolet lighting, identified to species, and counted. All marked and daily subsamples of unmarked females which were blood fed or gravid were isolated in vials for oviposition. Egg batches were held in the insectary for up to 48 h and then examined microscopically to determine the percent hatch. Unhatched, unembryonated egg batches were discarded. Fully hatched and unhatched or partially hatching embryonated egg batches were scored as D-13 ♂ or UM ♂ matings by the objective criterion established in the quality control section. Some partially hatched rafts were reared and the chromosomes of the emerging adults examined to verify the presence of the D-13 translocation.

Dissections.—Subsamples of UM ♀♀ and both D-13 and UM ♂♂ were dissected to determine reproductive status. For UM ♀♀, the spermathecae were examined for the presence of spermatozoa, the degree of ovarian development was scored by using the criteria of Mer (1936), and the number of previous ovipositions was determined by the number of dilatations on a subsample of individual ovariole tubules (Detinova 1962). Male age was determined by the number of spermatocysts and the relative proportion of the testes occupied by the sperm reservior (Mahmood and Reisen, unpublished data). Mating history (i.e., the number of previous ejaculations) was ascertained by the degree of engorgement of the accessory glands with secretory material and the degree of sperm depletion in the sperm reservoir and postgonadal system. Using these methods, it was not always possible to separate once-mated males, after a 36-h rejuvenation period, from virgin males of the same age. Thus, males were divided into two groups: (1) virgin, i.e., those unmated and once mated but having rejuvenated; and (2) mated, i.e., those once mated but not rejuvenated, and those mated more than once.

Calculations.—Recapture data analysis essentially followed that of Reisen et al. (1980a). Horizontal survivorship(s) was estimated by a least-squares fit of the number recaptured per man-hour per day (r_t) on time in days after release (t), where $\ln r_t = \ln a - \ln s(t)$ and a is the intercept of the regression function. The loss rate $L = 1 - s$. The number of released adults remaining within the study area on any day, $R_t = s(R_{t-1} + M_{t-1})$, where M_t = the number of marked mosquitoes released.

Mating competitiveness (*e*) was calculated by the method of Haisch (1970) as modified for mosquitoes by Grover et al. (1976a), where: $e = w/(1 - w) \cdot (1 - f)/f$; w = the number of UM ♂♂ among all ♂♂; and f = the number of UM ♂ matings among all determined matings based on egg batch fertility patterns. Formulae for the variance of e, $s^2(e)$, and for testing for significance between experiments by chi-square test followed those of Grover et al. (1976a). Competitiveness estimates were tested for significant departure from 1 by using a one-way chi square, with the expected frequency of fertile rafts calculated from *w*.

Results

Production, Release, and Quality Controls

A total of 10,203 D-13 ♂ pupae were counted and chemosterilized from 31 March to 5 April, of which only 75.7% sucessfully emerged through the self-marking device at Kot Baghicha (Table 1). Significantly less D-13 ♂♂ emerged than did SAT and KB ♀♀ released in identical self-marking devices ($\chi^2_{(1)} = 325.42$; $P < 0.001$). More than 90% of the adult females transported

Table 1.—Summary of release and recapture of D-13 ♂ pupae and SAT ♀♀ and KB ♀♀ released as pupae and 1- and 4-day-old adults

Genotype	Stage	Age (days)	Release date	No. produced	No. released	% Released	No. recaptured (♀♀-♂♂): Sheds C 1–5	Shed D	Pit shelter	Swarm	Total recaptured	% Re-captured	Loss rate
D-13 ♂♂	Pupa	—	1–5 April	10,203	7,727	75.7	0–317	0–127	0–54	0–320	0–118	10.6	0.450
SAT ♀♀	Pupa	—	3 April	2,247	1,875	83.4	113–0	47–0	7–0	0–0	167–0	8.9	0.336
	Adult	1	5 April	1,378	1,336	97.0	237–0	7–0	0–0	4–0	248–0	18.6	0.445
		4	5 April	965	918	95.1	148–0	8–0	1–0	2–0	159–0	17.3	0.403
KB ♀♀	Pupa	—	3–4 April	3,330	3,019	90.7	200–4	84–0	15–0	3–0	302–4	10.1	0.122
	Adult	1	6 April	1,951	1,914	98.1	312–2	30–0	1–0	2–0	345–2	18.1	0.235
		4	5 April	467	429	91.9	73–1	1–0	2–0	2–0	78–1	18.4	0.364

to Kot Baghicha successfully dispersed from the release cages.

The following quality controls were incorporated into the present experiment.

(1) Genetic Sexing System. Five subsamples of 9,058 D-13 pupae produced for release were sexed and counted under a dissecting microscope; 12 (0.13%) were females. These females were tested for susceptibility to dieldrin and were found to exhibit mortality patterns similar to homozygous susceptibles. Thus, we concluded that the gene for dieldrin resistance had not crossed-off the Y-chromosome, but rather some unhatched eggs were included in the larval insecticide treatment-phase and did not succumb to the dieldrin.

(2) Fertility of UM ♂♂ × UM ♀♀. A total of 114 blood-fed or gravid females collected resting indoors at Kot Baghicha on 26 and 31 March 1980 oviposited after individual isolation; mean fecundity (±95% confidence limit) = 150.5 ± 4.4 eggs per female; mean percent fertility = 95.7 ± 5%, range = 77.4 to 100%.

(3) Fertility of Chemosterilized D-13 ♂♂ × SAT ♀♀. A total of 146 SAT ♀♀ which had been crossed en masse to chemosterilized D-13 ♂♂ oviposited batches of embryonated eggs; mean percent fertility (±95% confidence interval) = 0.37 ± 1.0%, range = 0.0 to 7.6%. Therefore, chemosterilization was successful and matings of D-13 ♂♂ could readily be distinguished from matings with UM ♂♂ based on egg-batch fertility.

(4) Laboratory Competitiveness. Chemosterilized D-13 ♂♂ which were sexed manually (i.e., not exposed to dieldrin) were forced to compete for SAT ♀♀ against SAT ♂♂ while confined in 3.7 liter carton cages held in the insectary. Females were then fed on a mouse, isolated individually in vials, and the resulting egg batches were counted for hatched and unhatched eggs. Two competitiveness tests were done with 50 and 130 adults of each genotype per cage. Test 1: fertile rafts 50% hatch, f = 18 fertile of 29 total batches = 0.621; e_1 = 0.611. Test 2: f = 19/37 = 0.514; e_2 = 0.945. There was no significant difference in the competitiveness estimates ($\chi^2_{(1)}$ = 0.583; $P > 0.05$). A pooled laboratory competitiveness value was then calculated (f = 37/66 = 0.561; e = 0.783; $s^2(e)$ = 0.045) which was not significantly different from e = 1 ($\chi^2_{(1)}$ = 0.97; $P > 0.05$). Thus, in this small sample D-13 ♂♂ were equally competitive for SAT ♀♀ against SAT ♂♂ under laboratory conditions in small cages.

(5) Confirmation of D-13 ♂ Matings in Nature. Egg batches from UM ♀♀ with less than 10% fertility were reared individually, and the emerging adult males were examined cytologically for the presence of the translocation. One of five families from UM ♀♀ and two of five families from SAT and KB ♀♀ were confirmed as having mated with D-13 ♂♂; the remainder had mated with UM ♂♂ and represented sterility related to female oviposition difficulty in vials, handling injuries, or rare low fertility. However, most matings with D-13 ♂♂ produced insufficient larvae for cytological confirmation, and thus the few confirmations were extremely biased for UM ♂♂ matings.

Table 2.—Summary of female dissections during April 1980

Site	Mark status[a]	No. dissected	% Insemi-nated[b]	Trophic status (%)[c]				Dilatations[d]						
				NF[b]	PF	HG	G	0[b]	1	2	3	4	5	6
Indoors	UM	197	81.2a	17.8b	3.6	40.1	38.6	90a	53	15	8	3	4	2
Pit shelter	UM	60	5.0b	93.3a	3.3	3.3	0.0	57b	3	0	0	0	0	0
	M	19	0.0b	100.0a	0	0	0	19b	0	0	0	0	0	0

[a] UM, Unmarked; M, marked (KB + SAT).
[b] Proportions followed by the same letter were not significantly different when tested by a 2×2 contingency chi square ($P > 0.05$).
[c] NF, not fed (orarian stages N to Ib); PF, partially fed (stages Ib to IIa); HG, half gravid (stages III to IV); G, gravid (stage V). Ovarian stages as reported by Mer (1936).
[d] Dilatations of 22 UM ♀♀ resting indoors were not determined.

Recapture and Dissections.—Recapture success was less for adults released as pupae near a breeding site than as adults released inside indoor resting sites (Table 1). Alternatively, more females released as pupae were recaptured resting outdoors in the pit shelter and at shed D, than were females released indoors, indicating that mature females rest indoors near the site of feeding. The loss rates for females released as pupae after blood feeding and resting indoors were consistently lower than the loss of rates of females released in cattle sheds as adults (Table 1). Differences here were attributed to age and the intensity of removal sampling or both.

After emerging at the breeding site, adults seemed to rest outdoors for 1 to 2 days before being collected resting indoors or at male swarms. Proportionately more females resting in the pit shelter were uninseminated, unfed, and nulliparous than were females collected resting indoors (Table 2). Males also rested outdoors initially, since the proportion of unmarked males among all males (w) was first depressed at the pit shelter, then at the swarms and indoor resting sites at shed C, and finally at shed D (Table 3).

Less than 15% of the males collected resting outdoors had mated, i.e., ejaculated (Table 4). Exophilic resting young males presumably immigrated into the shed C compound where they were collected swarming and observed pairing. Swarming males were always more than 1 day old, and few males mated before 2 days of age. The proportion of males collected resting indoors which were mated was somewhat less than that observed at swarms but always exceeded the proportion resting outdoors. D-13 ♂♂ appeared to mate as frequently as the UM ♂♂, since the proportion mated in each collection method was not significantly different from the proportion of UM ♂♂ which had mated.

The chronological and reproductive aging of males was considered valid in the present study because the age structure of D-13 ♂♂ dusted blue, a date-specific mark, was similar to that of the UM ♂♂ (Table 4). In addition, we thought that our separation of mated from unmated males was valid, since seven UM ♂♂ with female-color fluorescent dust on their terminalia were scored as having mated. More pink-dusted D-13 ♂♂ were dissected later in the study when the number of blue-dusted D-13 ♂♂ diminished, and thus they were more heavily represented in the older age classes.

Competitiveness in Nature.—The competitiveness of D-13 ♂♂ in nature was assessed by using marked SAT ♀♀ or KB ♀♀ released as pupae or virgin adults and UM ♀♀ (Table 5). Females were considered to have mated during the two successive nights after the night of emergence or on the night of adult release. A pooled w was calculated based on male collections for these 2 days. For the UM ♀♀, the pooled w was calculated from collections on 2 to 11 April, since the exact nights of mating could not be ascertained. In the competitiveness calculations, only males and females collected resting indoors at shed C compound were used. Estimates of w based on swarm collections closely paralleled estimates calculated from indoor resting sites (Table 3), whereas estimates from the pit shelter were considered

Table 3.—Number of D-13 ♂♂ released and daily estimates of w (the proportion of UM ♂♂ among all ♂♂) calculated from pit shelter, swarm and indoor resting collections

Date	No. of D-13 ♂♂ released	w			
		Pit shelter	Swarm	Indoors: Shed C	Indoors: Shed D
31 March	272	—	—	—	—
1 April	2,405	0.941	1.000	1.000	1.000
2 April	2,839	0.963	1.000	0.996	1.000
3 April	1,132	0.571	0.983	0.942	1.000
4 April	826	0.842	0.886	0.886	0.954
5 April	253	0.425[a]	0.872	0.841	0.909
6 April		0.552	0.747[a]	0.734[a]	0.874
7 April		0.867	0.818	0.758	0.828[a]
8 April		0.950	0.846	0.855	0.903
9 April		1.000	0.933	0.951	0.951
10 April		0.981	0.952	0.974	0.940

[a] Maximal depression of w in each collection series.

Table 4.—Summary of male dissections at Kot Baghicha during April 1980[a]

		Genotype (mark status)					
		UM ♂♂		D-13 ♂♂ (pink)		D-13 ♂♂ (blue)	
Location	Age (days)	V	M	V	M	V	M
Pit shelter	0	23	0	2	0	1	0
	1	12	1	2	0	0	0
	2	7	0	1	0	1	0
	3–5	9	3	3	0	1	1
	5–8	3	2	0	1	0	0
	>8	2	3	0	0	0	0
	Total	56	9	8	1	3	1
	M (%)[b]	13.8a		11.1a		25.0NT	
Swarm	0	0	0	0	0	0	0
	1	4	0	1	0	0	0
	2	3	0	0	0	5	3
	3–5	5	10	0	1	6	11
	5–8	3	3	3	9	0	4
	>8	3	3	7	7	0	0
	Total	18	16	11	17	11	18
	M (%)[b]	47.1b		60.7b		62.1b,c	
Indoor resting	0	2	0	0	0	0	0
	1	11	1	3	0	9	1
	2	2	2	0	0	9	5
	3–5	10	7	0	0	12	8
	5–8	4	4	6	8	3	1
	>8	4	3	6	5	0	0
	Total	33	17	15	13	33	15
	M (%)	34.0b		46.4b		31.3b, d	

[a] Numbers of UM ♂♂ or D-13 ♂♂ marked pink (released on 31 March and 2 to 5 April) and blue (released on 1 April) that were classified as virgins (V) or mated (M).
[b] Percent M = mated/total × 100. Values followed by the same letter in rows or columns were not significantly different when compared by a 2×2 chi-square test ($P > 0.05$). NT, too few to test.

inappropriate since the males were not yet sexually mature (Table 4).

Competitiveness estimated by different release procedures was compared by chi square (Grover et al. 1976a) (Table 5). Estimates based on SAT ♀♀ and KB ♀♀ which were 1 and 4 days of age when released inside resting sites were statistically similar throughout ($P > 0.05$), indicating the age of the virgin females at release was inconsequential. Data were pooled and then compared for within-genotype competitiveness estimated for females released as pupae (*P*) and adults (A). Results were mixed; SAT-P was significantly greater than SAT-A, while KB-P was significantly less than KB-A (Table 5). However, when data were pooled by release method, there was no significant difference in overall competitiveness (Pupal release $e = 0.277$; adult release $e = 0.425$; $\chi^2_{(1)} = 1.43$; $P > 0.05$). Thus, at this point we have no data to indicate differences in male competitiveness when the females used for assessment are released either as pupae at a breeding site or as adults of different ages inside diurnal resting sites.

Pooled competitiveness estimates calculated for SAT ♀♀ and KB ♀♀ did not differ significantly ($P > 0.05$) when tested by chi square (Table 5). The pooled SAT ♀♀ estimate in nature was also not significantly different from the pooled SAT ♀♀ laboratory estimate or the estimates for UM ♀♀ in nature. These results indicate that assortative mating had not been induced by

Table 5.—Field and laboratory mating competiveness estimated for D-13 ♂♂, using SAT laboratory strain ♀♀, laboratory-reared ♀ progeny of KB ♀♀ and endemic UM ♀♀[a]

		Males				Females			Competitiveness[c]				
						Matings							
Female genotype	Mating date, 1980[b]	D-13 R_t	Σ D-13	Σ UM	*w*	D-13	UM	*f*	*e*	$s^2(e)$	*e* = 1, $\chi^2_{(1)}$	Pooled *e*	*e* = 1, $\chi^2_{(1)}$
SAT (LAB)	—	—	180	180	0.500	29	37	0.561	0.783	0.044	0.97NS	0.783a	
SAT-P	4–5 April	1,182	97	618	0.864	4	41	0.911	0.621	0.110	0.84NS	0.385ab	16.39*
SAT-A	5–6 April	946	121	430	0.780	14	190	0.931	0.263	0.006	27.27*		
KB-P	4–6 April	1,066	159	789	0.832	4	101	0.962	0.196	0.010	12.63*	0.580a	10.12*
KB-A	5–7 April	809	181	618	0.773	33	185	0.849	0.606	0.016	7.12*		
UM	2–11 April	571	313	2,659	0.895	49	1,368	0.965	0.309	0.002	74.78*	0.309b	

[a] See text for explanation of strains.
[b] SAT (LAB) = combined laboratory competitiveness, using SAT ♀♀ vs. SAT ♂♂; SAT-A and KB-A combined 1- and 4-day-old adult estimates which were not significantly different when tested by $\chi^2_{(1)}$. SAT-P and KB-P released as pupae.
[c] $e = 1$, $\chi^2_{(1)}$: chi-square testing for equal competetiveness, $e = 1$, based on expected value *w* (NS = nonsignificant, $e = 1$, $P > 0.05$; asterisk indicates $e < 1$, $P < 0.01$). Pooled *e* followed by the same letter(s) not significantly different when tested by $\chi^2_{(1)}$ ($P > 0.05$).

laboratory colonization or maintenance. Disappointingly, pooled field competitiveness estimates were significantly less than 1 when tested by a one-way chi square, using w as the expected proportion of UM ♂ matings among all matings. Thus, less D-13 ♂♂ were statistically uncompetitive in nature, regardless of the female genotype used for assessment.

Discussion

Chemosterilized D-13 ♂♂ behaved similarly to males from the Kot Baghicha population. After emergence from the self-marking device, D-13 ♂♂ first rested outdoors, selecting the same sites as UM ♂♂. After maturation, which included the full rotation of the terminalia, D-13 ♂♂ flew to the shed C cattle compound, where they swarmed with UM ♂♂ and mated with SAT, KB, and UM ♀♀. The proportions of D-13 ♂♂ and UM ♂♂ which had mated were comparable statistically in swarm and resting collections. These results were encouraging, since previously released males carrying a chromosomal aberration were reproductively competitive for SAT and KB ♀♀ but rarely inseminated UM ♀♀ (Baker et al. 1980).

The male dissection data suggested that some males (both D-13 and UM) do not mate during their lifetime, whereas others appeared reproductively depleted and may have participated in four or more matings. Thus, the percentage of males contributing to the population gene pool may be restricted to only a limited proportion of the total male population which may be more sexually aggressive. We attributed the increased number of UM ♀ matings during the present experiment to the release of D-13 ♂♂ as pupae near a suitable breeding site which simulated the natural condition. However, it was not possible to ascertain exactly where the inseminations occurred, although the probable location was either near the exophilic resting site or at swarms near the diurnal, cattle shed resting sites. Interestingly, swarming and mating were never observed near the breeding site, near confirmed exophilic resting sites, or near a cattle shed in a recently vacated compound (formerly shed E in Baker et al. 1980). Alternatively, the higher mating success with UM ♀♀ during the present study may have simply reflected the higher emergence rate and greater proportion of uninseminated females resting in shed C compound than in our previous experiment.

Despite encouraging similarities in resting, swarming, and mating behavior, coupled with possible improvements in release strategies, D-13 ♂♂ mated with a disproportionately low number of SAT, KB, and UM ♀♀ and must be classified as less competitive. Speculative explanations include: (1) loss of vigor due to dieldrin and/or chemosterilant exposure; (2) handling stress associated with production, transport, and release; and (3) alterations in behavior and mating compatability related to the chromosomal rearrangement and laboratory-adapted genetic background. We have no data to indicate that the use of either dieldrin or bisazir was responsible for the poor competitiveness of the D-13 ♂♂. The gene for dieldrin resistence persists naturally in the Kot Baghicha gene pool and has been maintained for almost 10 years, since the last dieldrin spray. The proper use of chemosterilants does not seem to alter dispersal or longevity (e.g., Rajagopalan et al. 1973), and treated males have been used in successful population suppression attempts (e.g., Lofgren et al. 1974).

The rearing methods used during the present study for production were similar to normal insectary maintenance procedures and were not considered detrimental. Possible mechanical injuries to the pupae may have occurred during volumetric counting, when the pupae were tightly packed in the measuring cylinder, or during rinsing after chemosterilization. Additional losses may have occurred during emergence through the self-marking device at the study site. The reduced emergence success of the D-13 ♂♂ pupae did suggest that some handling injury may have occurred. We thought, however, that injured specimens were lost in the self-marking device and that adults healthy enough to fly to shed C compound (ca. 100 m) were probably in good enough condition to mate. Since w was estimated from the proportion of these males in the natural population, competitiveness estimates should not have been low if the males were competitive.

Further experimentation with D-13 ♂♂ is currently underway to assess laboratory mating competitiveness with and without dieldrin and chemosterilant treatment. The present overall laboratory competitiveness estimate with chemosterilization was 0.78 which was lower than desired, but not significiantly less than 1.0. We plan to improve D-13 ♂♂ laboratory competitiveness by altering the background genotype or improving chemosterilization procedures before subsequent experimentation.

Although our estimated field competitiveness for the chemosterilized D-13 ♂♂ was less than desired, our results were comparable to release experiments with other mosquito species. Grover et al. (1976a) estimated field competitiveness of chemosterilized *Culex quinquefasciatus* in India to be between 0.20 and 0.50, whereas estimates were 0.86 in laboratory cages and 0.79 in field cages. Weidhaas et al. (1974) estimated competitiveness of male *A. albimanus* during a successful suppression in El Salvador to be between 0.25 and 0.5 based on production and population reduction statistics. Higher competitiveness values have been attained for more "domestic" *Aedes aegypti*, using similar experimental design (e.g., Grover et al. 1976b). Competitiveness estimates for unmarked females may be reduced by the immigration of already inseminated females from neighboring micropopulations and by the survival of older females inseminated before the release of the sterile males. Due to the high degree of intermicropopulation exchange in the Kot Baghicha area (Reisen et al. 1980b), planned control attempts will incorporate the use of an insecticide boundary to isolate the target population.

Acknowledgment

We especially thank J. Ahmad, S. Amar, S. H. Chishti and T. J. Rathore, who assisted with the collections, and M. Saghir, N. Hussain, M. Tahir, L.

Chowdhry, Z. Ahmad, R. Yamin and M. R. Sindhoo, who assisted with production and assessment. This research was funded by grants AI-10049 and AI-16289, National Institute of Allergy and Infectious Diseases, N.I.H., and PL-480 grant 391-0455 from the United States Agency for International Development, Pakistan.

REFERENCES CITED

Ainsley, R. W. 1976. Laboratory colonization of the malaria vector, *Anopheles culicifacies*. Mosq. News 36: 256–258.

Akhtar, R., A. Learmouth and M. Keynes. 1977. The resurgence of malaria in India 1965–1967. Geojournal 1: 69–79.

Bailey, D. L., R. E. Lowe, D. A. Dame, and J. A. Seawright. 1980. Mass rearing the genetically altered MACHO strain of *Anopheles albimanus* Wiedemann. Am. J. Trop. Med. Hyg. 29: 141–149.

Baker, R. H., W. K. Reisen, R. K. Sakai, H. R. Rathor, K. Raana, S. Niaz, and K. Azra. 1980. *Anopheles culicifacies* Giles: mating behavior and competitiveness in nature of males carrying a complex chromosomal aberration. Ann. Entomol. Soc. Am. 73: 581–588.

Baker, R. H., R. K. Sakai, and K. Rana. 1981. Genetic seking for a mosquito sterile male release. J. Hered. (in press).

Davis, E. W., and D. J. Gould. 1973. A portable suction apparatus for collecting adult mosquitoes. Mosq. News 33: 246–247.

Detinova, T. S. 1962. Age-grouping methods in Diptera of medical importance. W. H. O. Monogr. 47: 1–216.

Grover, K. K., C. G. Curtis, V. P. Sharma, K. R. P. Singh, D. Dietz, H. V. Agarwal, R. K. Razdan, and V. Vaidyanathan. 1976. Competitiveness of chemosterilized males and cytoplasmically incompatible translocated males of *Culex pipiens fatigans* Wiedemann (Diptera, Culicidae) in the field. Bull. Entomol. Res. 66: 469–480.

Grover, K. K., S. G. Suguna, D. K. Uppal, K. R. P. Singh, M. A. Ansari, C. F. Curtis, D. Singh, V. P. Sharma and K. N. Panicker. 1976b. Field experiments on the competitiveness of males carrying genetic control systems for *Aedes aegypti*. Entomol. Exp. App. 20: 8–18.

Haisch, A. 1970. Some observations on decreased vitality of irradiated Mediterranean fruit fly. *In* Sterile-male technique for control of fruit flies. IAEA, Vienna, pp. 71–75.

Lofgren, C. S., D. A. Dame, S. G. Breeland, D. E. Weidhaas, G. Jeffery, R. Kaiser, H. R. Ford, M. D. Boston, and K. F. Baldwan. 1974. Release of chemosterilized males for the control of *Anopheles albimanus* in El Salvador. III. Field methods and population control. Am. J. Trop. Med. Hyg. 23: 288–297.

Manouchehri, A. V., A. Zaini, and E. Javadian. 1975. Resistance of *Anopheles culicifacies* Giles to DDT in Baluchistan Province, Southern Iran, 1974. Mosq. News 35: 324–316.

Mer, G. G. 1936. Experimental study on the development of the ovary in *Anopheles elutus*, Edw. (Dipt. Culicidae). Bull. Entomol. Res. 27: 351–354.

Muirhead-Thompson, R. C. 1958. A pit shelter for sampling outdoor mosquito populations. Bull. W.H.O. 19: 1116–1118.

Mukhtar, R., T. F. Siddiqui, and W. K. Reisen. 1980. Rearing experiments for producing large numbers of *Culex tritaeniorhynchus* Giles. Pak. J. Zool. (in press).

Rajagopal, R. 1977. Malathion resistance in *Anopheles culicifacies* in Gujarat. Indian J. Med. Res. 66: 27–28.

Rajagopalan, P. K., M. Yasuno, and G. C. LaBrecque. 1973. Dispersal and survival in the field of chemosterilized, irradiated and cytoplasmically incompatible male *Culex pipiens fatigans*. Bull W.H.O. 48: 631–635.

Reisen, W. K., M. Aslamkhan, M. Suleman, and Z. A. Naqvi. 1976. Observations on the diel activity patterns of some Punjab mosquitoes (Diptera: Culicidae). Biologia 22: 67–77.

Reisen, W. K., F. Mahmood, and T. Parveen. 1980a. *Anopheles culicifacies* Giles: a mark-release-recapture experiment with cohorts of known age with implications for malaria epidemiology and genetical control in Pakistan. Trans. R. Soc. Trop. Med. Hyg. 74: 307–317.

Reisen, W. K., F. Mahmood, and K. Azra. 1980b. *Anopheles culicifacies* Giles: adult ecological parameters measured in rural Punjab Province, Pakistan, using capture-mark-release-recapture and dissection methods, with comparative observations on *An. stephensi* Liston and *An. subpictus* Grassi. Res. Popul. Ecol. (in press).

Singh, N., and M. Yasuno. 1971. A device for self-marking mosquitoes. WHO/VBC/71.330. (unpublished working document).

Yasuno, M., P. K. Rajagopalan, S. Russell, and G. C. LaBrecque. 1972. Dispersal of *Culex fatigans* in Delhi villages. WHO/VBC/72.352/13 pp. (unpublished working document).

Weathersby, A. B. 1963. Harvesting mosquito pupae with cold water. Mosq. News 23: 249–251.

Weidhaas, D. C., S. G. Breeland, C. S. Lofgren, D. A. Dame, and R. Kaiser. 1974. Release of chemosterilized males for the control of *Anopheles albimanus* in El Salvadore. IV. Dynamics of the test population. Am. J. Trop. Med. Hyg. 23: 298–308.
