# Supplementary material for: Mark-release-recapture of male Aedes aegypti (Diptera: Culicidae): Use of rhodamine B to estimate movement, mating and population parameters in preparation for an incompatible male program
Source: PLoS Negl Trop Dis. 2021 Jun 7;15(6):e0009357. doi: 10.1371/journal.pntd.0009357 (PMC8183986; doi:10.1371/journal.pntd.0009357)
Supplement: Attachment — Submitted filename: Response to reviewers-Final.docx [file pntd.0009357.s007.docx]

**Response to reviewers**

We would like to thank both reviewers for their comments and suggestions on ways to improve our manuscript and we appreciate the time taken to provide this feedback. Their comments have certainly spurred some deep thinking on our part about what assumptions underlie each of the models used to estimate results. While there have been some changes to methods and results as an outcome of this, we believe these have strengthened the manuscript without having a major impact on conclusions. Please find responses to reviewer in-text comments below. All line numbers are related to the current manuscript where no edits are visible.

**Methods**

**Reviewer #1: The MRR methods are well done and clearly presented. Several aspects should be considered:**

**1. LPI: Because male survival was so low, n1 should be adjusted to account for daily losses due to emigration and death. n2 is presumed constant with losses = additions.**

We would like to thank the reviewer for the many suggestions which have led to considerable discussion around the use of the LPI in a mosquito system. Historically, the Lincoln Peterson Index and modifications assume a closed population and do not take into consideration survival of released individuals. We would like to acknowledge that the low survival of Ae. aegypti males would tend to bias population estimates over longer periods. To compensate for this and to take into consideration the reviewers comment, we have recalculated the LPI population estimates by only selecting those wild and marked males caught before the average life expectancy (which is calculated from daily survival) and only for MRRs 3, 4, & 5 where full mixing within the population was ensured.

Please see Methods, paragraph 8, lines 201-203:

“To account for the low survival of released Ae. aegypti males, only marked males captured the periods before the ALE (rounded up) of each experiment were selected.”

**2. Mating competitiveness. There are published methods to estimate male mating competitiveness [see Grover et al. 1976 Bull Ent Res 66: 469-480 and for aegypti Ent Exp. Appl. 20: 8-18; an example of their use is attached]. The authors have all the necessary data to estimate the performance of the released males and should provide these metrics as they will indicate the degree of 'over-flooding' necessary.**

We would like to thank the reviewer, again, for bringing to our attention an estimate of competitiveness that involves ratios of released and mated individuals. As such we have included this in the manuscript to show how mating competitiveness varied between experiments. Results suggest that there was no significant difference between marked and wild males across all experiments. As these experiments did not involve the use of Wolbachia infected males, an estimate of population over-flooding was not required.

**3. Dispersal. Comparing single vs transect releases was difficult because the authors could not tell from where males were released when recaptured during the transect release. This was discussed, but could be made more clear.**

We have included further detail in the methods regarding the multipoint kernel estimates. It should be noted that there is no statistical issue with inference for a multi-point release where the release location of the trapped individuals is unknown. If different coloured markings were used and this information were available, the dataset would be more informative and we would certainly expect to get better estimates, but it is certainly possible to perform the inference without this. We have included extra detail in the methods lines 224-228:

“Multi-point releases treat the trapping intensity at each site as a finite mixture of the dispersal kernels from each release point, so the unknown latent variable of release location for each trapped mosquito has been integrated out of the probability density function used for the likelihood equation.”

For single point releases we have provided detail previously:

Trewin BJ, Pagendam DE, Zalucki MP, Darbro JM, Devine GJ, Jansen CC, et al. Urban Landscape Features Influence the Movement and Distribution of the Australian Container-Inhabiting Mosquito Vectors Aedes aegypti (Diptera: Culicidae) and Aedes notoscriptus (Diptera: Culicidae). Journal of Medical Entomology. 2019;57(2):443-53.

**4. Female age structure. It was not clear if the BGS traps collect mostly unfed/newly emerged females resulting in the very high proportion of uninseminated females reported. Perhaps the authors could briefly indicate the proportions of unfed, blood fed and gravid females collected in the Results section as this would affect the insemination rate? Was there any adult mosquito control that would eliminate the older portion of the wild population?**

Female age structure was not something examined during this experiment as abdomens were destroyed during the dissection of the female spermathecae. We also did not examine whether adult females were unfed, blood fed or gravid. We note that work on BGS traps in north Queensland indicate (without the use of CO^2^ or lures), show a slight bias towards collecting blood-fed parous females. From the work of Ball et al 2010:

“Biases were not detected across age groups or body sizes. A significant bias was detected across physiological groups: nulliparous females were recaptured at a significantly lower rate than all other groups except blood-fed parous females, which were also recaptured at a low rate by the BGS.”

Ball, T. S., & Ritchie, S. R. (2010). Sampling biases of the BG-sentinel trap with respect to physiology, age, and body size of adult Aedes aegypti (Diptera: Culicidae). Journal of medical entomology, 47(4), 649-656.

Mosquito control in north Queensland only occurs within 100m of an imported or local dengue case. As there were no dengue cases in the areas during experiments, so no mosquito control was being undertaken in the region.

**Editorial and Data Presentation Modifications?**

**Reviewer #1: Although generally well-prepared, I thought the paper will need some revision to improve clarity. Edits were made directly on the attached manuscript using tracked changes. The authors love to use abbreviations and these should be carefully presented so that the reader does not get lost in the 'alphabet soup'.**

Where necessary we have provided further clarification for abbreviations. The majority of these were first defined in the introduction, with only minimal number of new abbreviations introduced in the methods. For clarity we can define all abbreviations in the methods section if required.

**Reviewer #2: Recommend making data and code available in a permanent digital repository (e.g., Figshare or Scientific Data) or as supplementary files with the manuscript.**

Thank you for the suggestion of a permanent digital repository. We have now created an extra repository for the STEIK R package alongside the mosquito recapture data at the CSIRO Data Access Portal where they now have permanent DOIs.

Further information on the CSIRO DAP:

The Australian Commonwealth Scientific and Industrial Research Organisation (CSIRO) was created in 2016 and is Australia's national science agency, one of the largest and most diverse research agencies in the world. CSIRO is committed to ensuring its online content is accessible to everyone regardless of ability. The CSIRO Data Access Portal (DAP) provides access to research data, software and other digital assets published by CSIRO across a range of disciplines. The DAP is maintained by CSIRO Information Management & Technology and is a permanent archive for the preservation of research data assets retained for reference and reuse purposes.

<https://data.csiro.au/collections>

**Summary and General Comments**

**Reviewer #2: My comments are included in the attached PDF. Overall, I found this paper by Trewin et al. to be a very well-written, methodologically sound, and scientifically valuable contribution to the literature on Ae. aegypti movement. The information on spatial patterns of insemination by marked males is especially valuable given the growing interest in SIT/IIT approaches.**

**The biggest limitation of the paper as written is that it did not acknowledge two key recent papers on movement of Ae. aegypti females and males, which are indicated in my comments (Marcantonio et al. 2019 and Juarez et al. 2020). These papers trapped over broader spatial ranges than most earlier studies and utilized more rigorous modeling approaches (GLMMs and dispersal kernels) very similar to those used in this paper. This resulted in overstatement of the novelty of this study's findings, and the manuscript would benefit from comparing and contrasting the North Queensland results to those of the other studies.**

We must thank the reviewer for bringing to our attention two recently published mark-release-recapture experiments on male Ae. aegypti. We have now incorporated these into the manuscript and ensured that we have changed our language to reflect how our results confirm these findings. Please see responses to in-text comments below for further detail.

**The use of Github and an institutional repository for the modelling code and data from this study are not ideal because there is no guarantee that they will persist into the future. These resources should be preserved as supplementary materials on the PNTDs website or in another permanent digital repository with a DOI.**

Thank you for the suggestion of a permanent digital repository. As mentioned above we have now created an extra repository for the STEIK R package alongside the mosquito recapture data at the CSIRO Data Access Portal where they now have permanent DOIs. This information could also be stored on the PNTD website if further desired by the reviewers or editors.

**Specific responses to in-text comments:**

**Reviewer 1:**

**Abstract**

**Abstract, Line 23:**

**Unclear how digital technologies control mosquitoes?**

This is a broad opening sentence describing new trends that are revolutionizing the biological control of vectors. We have changed digital technologies to “digital support systems”.

**Abstract, Line 32:**

**Seems high? Was the population abundance increasing?**

There is no evidence the population was increasing primarily due to differences in rainfall patterns between seasons. However, 40% daily could suggest consistent emergence and this could just be a feature of the landscape in north Queensland. There is evidence that suggested the population sizes were much different between the two periods studied due to differences in rainfall between each season (dry/wet).

Lines 421-430:

“As release numbers were relatively similar across all experiments, these results suggest that the proportion of total mated females remains relatively static regardless of the number of additional males added to a population and sterile to wild-type mating ratios will be proportional to the number of each in the population. This result is unlikely to be influenced by trapping bias (56) or a lack of mating competitiveness by rhodamine B marked males. Wild females are likely available for mating immediately upon release of marked males, with ~40% of daily females unmated by marked or wild-type males at any given time. This suggests that females emerged and became available for mating at a relatively constant rate throughout the study.”

**Abstract, Line 32:**

**By released males or no sperm in spermathecae period?**

No sperm at all in spermathecae. The sentence in the discussion (lines 439-440) has been modified for clarity:

“Wild females are likely available for mating immediately upon release of marked males, with ~40% of daily females unmated by marked or wild-type males at any given time.”

**Abstract Line 33:**

**Unclear? Forever?**

For clarity we have modified this to “multiple times per week”:

“Results showed that ~40% of wild females captured daily were unmated, suggesting interventions will need to release males multiple times per week to be effective at suppressing *Ae. aegypti* populations.”

**Introduction**

**Introduction, paragraph 4, lines 89-90:**

**Jargon – what do you mean by ‘over-flooding’???**

We have changed the sentence to provide a more description for the term. However, the use of “over-flooding ratio” has been used since the early days of SIT (see Knipling 1952). We believe it is necessary to introduce and use this term when describing the challenge of estimating population sizes and how MRRs can assist in determining important biological parameters when estimating over-flooding ratios.

New sentence:

“The primary challenge for SIT/IIT strategies is the determination of adequate release numbers to supplant matings with wild males (or “over-flooding ratio”) across large areas.”

**Methods**

**Methods, paragraph 2, lines 149-153:**

**So you can’t report number of generations in colony?**

We have included further detail to the number of generations in colonies before releases occurred:

*“**Aedes* *aegypti* colonies were newly established with wild type eggs collected from multiple ovitraps in Innisfail before each experimental period. Mosquito colonies were maintained using standard laboratory rearing protocols with 28 °C ± 1 °C, a 70 % (± 10 %) relative humidity, and a 12:12 hour light cycle and twilight period. F1 – F3 generation wild-type *Ae. aegypti* larvae were hatched into a solution of 0.2 g/L yeast in water in which they were allowed to feed for 24 hours.”

**Methods, paragraph 5, lines 180-181:**

**Unclear? With a single point release, males would be concentrated at near the release point?**

From previous experiments we expected males to move through the landscape and by delaying trapping we provided a greater period for which to disperse and mate with females without being captured in traps. For clarity we have modified to the sentence to include marked males.

“For MRR 1 and 2, traps were turned on after 24 hours to allow for mixing of marked males with the wild population.”

**Methods, paragraph 6, lines 183-184:**

**For how many days?**

We have amended the text to describe how long traps remained on. We have also included the trapping period in Table 1.

“All traps were serviced daily throughout each MRR experiment until no marked males were present in the sample.”

**Methods, paragraph 6, lines 186-187:**

**Some matings are not successful?**

We have changed the word to inseminated.

“Females were considered to be inseminated by a released marked male if rhodamine B was observed in the bursa, spermathecae or both.”

**Methods, paragraph 6**

**Could you determine if the female had mated with a wild and a released male?**

Yes this is possible, but it occurs rarely. It can occur only if the second mating occurs after the transfer of sperm from the first mating to the spermathecae, but before the female is refractory to a second mating. When this happens, the seminal fluid and sperm from the second mating most often do not transfer to the spermathecae. The presence of unmarked sperm in the spermathecae but presence of rhodamine B in the bursa would indicate a mating first by a wild female and then another by a marked male, or vice versa if the pattern was reversed. In previous work we have demonstrated that there is no leakage or transfer of Rhodamine B to previously wild mated females when marked males where introduced (in bugdorms) 24 h after the first mating.  This is supported by observations by Degner and Harrington (AJTMH, 2016) that state “Most females became refractory to a second mating within 2 hours after mating, and rates of polyandry ranged from 24% immediately after mating to 3% at 20 hours after mating.” Thus, multiple matings must occur in quick succession, but if they happen too quickly we could not tell the difference between a single or polyandrous female. When everything is considered, the data suggests that the rate and subsequent influence of polyandrous females on our results would be minimal.

**Methods, paragraph 7, line 191:**

**Don’t use undefined initials. Spell out when first used?**

We have defined PDS as the probability of daily survival in this sentence.

“For all experiments the probability of daily survival (PDS) was estimated by regressing log10 (x+1) the number of recaptured males against days since release, where the antilog10 of the regression slope was the PDS (39).”

**Methods, paragraph 7, line 193:**

**Why use natural logs when you initially used log base 10?**

The natural log and log base 10 transformation has been used extensively in the literature to calculate the probability of daily survival and the average life expectancy from the regression of the number of recaptured individuals over time. For the regression calculation of the PDS please see:

Gillies M. Studies on the dispersion and survival of Anopheles gambiae Giles in East Africa, by means of marking and release experiments. Bull. Entomol. Res. 1961;52(01):99-127.

For the ALE transformation please see:

Niebylski M, Craig Jr G. Dispersal and survival of Aedes albopictus at a scrap tire yard in Missouri. J. Am. Mosq. Control Assoc. 1994;10(3):339-43.

Walker, E. D., Copeland, R. S., Paulson, S. L., & Munstermann, L. E. (1987). Adult survivorship, population density, and body size in sympatric populations of Aedes triseriatus and Aedes hendersoni (Diptera: Culicidae). *Journal of medical entomology*, *24*(4), 485-493.

And first use:

Macdonald, G. (1957). The epidemiology and control of malaria. The Epidemiology and Control of Malaria.

**Methods, paragraph 8, lines 201-203:**

**Shouldn’t this be adjusted by daily survivorship, especially since male life expectancy was short [see above].**

While the Lincoln Peterson Index and modifications assume a closed population we acknowledge that the low survival of *Ae. aegypti* males would tend to bias population estimates over longer periods. To compensate for this, we have recalculated the LPI population estimates by only selecting those wild and marked males caught before the average life expectancy calculated for MRRs 3, 4, & 5.

“To account for the low survival of released *Ae. aegypti* males, only marked males captured the days before the ALE (rounded to the nearest whole number) of each experiment were selected.”

**Methods, paragraph 8, lines 193-201:**

**Depends on which traps were used for the estimates, why else develop the dispersal kernels?**

In this paper we have used two “models”: (i) dispersal under an assumption of a simple movement Brownian motion movement model; and (ii) the Lincoln-Peterson index (LPI), which is underpinned by a set of assumptions that we could regard as a latent model.  These two models are used for different purposes, the first is used to estimate mean dispersal distances (amongst other things) and the second is used to estimate population sizes.  We acknowledge that the core assumptions of these two models are incongruent in that the LPI assumes a well-mixed, closed population, whilst our dispersion model does not.  There is no easy way to reconcile these two “models”.  All we can do is acknowledge that each model we have used is based on a set of assumptions and that in almost all models, these assumptions are wrong (a model being a simple abstraction of the real world), but that the outputs of the models may still be useful (paraphrasing George Box).

We thank the reviewer for thinking about these issues and it has certainly spurred some deep thinking on our part about what assumptions underlie each of these models.  Since there is no way to have congruence between the LPI and out dispersion model, we have opted to recompute the LPI using only the data from our multi-point release MRR experiments (MRR 3, 4 & 5) and to only use traps that were proximal to the release locations.  Our rationale here is that the released individuals could be more readily assumed to have adopted the same spatial distribution as the wild/unmarked mosquitoes in a heterogeneous landscape (i.e. the released individuals are considered well-mixed) in the areas around these traps compared to at locations distal to the release location.

“The Lincoln Peterson Index with Chapman modifier was used to estimate population size (41). Where N ̂ is the size of the population, n_1_ is the number of marked animals released into the population, n_2_ the total number of individuals captured (marked and unmarked), and m_2_ the total number of marked individuals recaptured:

$$\hat{N}=\frac{\left( n_{1}+1 \right){(n}_{2}+1)}{(m_{2}+1)}-1$$

To account for the assumption that marked insects become completely mixed within the local population, only males from the release block and from multi-point (MRR 3 & 5) or the linear releases were selected for analysis (MRR 4).”

**Methods, paragraph 8, lines 201-202:**

**How was this determined?**

Before these experiments were undertaken, very little was known about the movement of male Ae. aegypti. What was known suggests that the species did not like to cross movement barriers such as roads, forests and open grass areas. Part of this experiment was designed to test these assumptions. In regards to the Lincoln Peterson assumptions, no study meets these with absolute certainty, and steps need to be undertaken to ensure these assumptions aren’t violated. For the fifth assumption, that there is little immigration and emigration, we chose a location with considerable movement barriers to the west (a large road), the east (a forest) and roads which would act as barriers elsewhere. This was to ensure minimal numbers of males and females would enter the release block where the majority of traps were located, and where estimates of population size were made.

**Methods, paragraph 8, lines 208-209:**

**Define corrections for trapping distance vs density**

We have included further detail on the use of annuli for distance:

“Traditional methods of MDT were calculated using the methods of Lillie et al (29) and Morris et al (28) where annuli are drawn around the release point to estimate dispersal distances:”

**Methods, paragraph 9, lines 215-217:**

**unclear**

We have added extra detail to the sentence to describe how the 50% and 90% flight ranges are estimated.

“Flight range (FR) of male movement was estimated from the linear regression of the cumulative ERs for each annulus (x axis) on the log_10_ (y axis) as the value of the y axis at 50% (FR_50_) and 90% (FR_90_), of the largest value of the x axis (13).”

**Methods, paragraph 10, lines 236-241:**

**Why not use these kinds of data to estimate the mating competitiveness of released males by comparing marked vs unmarked males in traps with females with and without marked sperm?**

We have incorporated a mating competitiveness estimate for all MRR experiments in the manuscript (see results Table 1 Lines 290-292). The estimate for competitiveness is incorporated below:

“Mating competitiveness was estimated using the methods of Reisen et al. (44) where the mating competitiveness (C) was calculated from the number of unmarked males among all males (w); and the number of unmarked male matings among all determined matings (f) where:

C= w/((1-w) ) × ((1-f))/f

The variation and test for significance between experiments was via chi-squared test following Grover et al. (45).”

**Results**

**Results, paragraph 2, lines 285-293:**

**Because the experiments were relatively close together, how did you separate recaptures from each experiment?**

In laboratory experiments by Johnson et al 2017 where our protocol was developed, wild-type male *Ae. aegypti* from north Queensland rarely survived longer than four days in large outdoor cages with or without the rhodamine B mark. This is consistent with other studies of male lifespan (see references below). Although each season’s experiments were conducted relatively closely together, a delay of seven-to-nine days between each release allowed us to assume that the majority of marked males had died. It is also assumed that the rhodamine B mark would no longer be detectable in males over the age of a week.

Maciel‐de‐Freitas, R., Codeco, C. T., & Lourenço‐de‐Oliveira, R. (2007). Body size‐associated survival and dispersal rates of Aedes aegypti in Rio de Janeiro. Medical and Veterinary Entomology, 21(3), 284-292.

Muir, L. E., & Kay, B. H. (1998). Aedes aegypti survival and dispersal estimated by mark-release-recapture in northern Australia. The American journal of tropical medicine and hygiene, 58(3), 277-282.

Fouque, F., Carinci, R., Gaborit, P., Issaly, J., Bicout, D. J., & Sabatier, P. (2006). Aedes aegypti survival and dengue transmission patterns in French Guiana. *Journal of Vector Ecology*, *31*(2), 390-399.

**Results, paragraph 2, line 287-288:**

**But not significantly so?**

This was not a significant difference (see error bars Fig 2), however, we have included the term “generally” as suggested to describe the trend.

**Results, Figure 2, lines 295-297:**

**Mean per trap or total per day averaged across experiments**

Figure 2 displays a daily aggregate and the second sentence in the caption describes the experimental period over which the data was aggregated.

“Daily mean recapture rate (±SD) of rhodamine B marked male (blue circles) and inseminated female Aedes aegypti (purple squares) in South Innisfail, Australia. Data aggregated across all mark-release-recapture experiments.”

**Results, paragraph 3, line 308:**

**Not shown in Table 2**

We have rewritten this sentence.

“Estimates of survival and ALE varied considerably for each experiment (Table 2).”

**Results, paragraph 3, line 311-313:**

**Total or unmarked?**

We have redone this calculation and clarified this by adding “wild” male population.

“Estimated wild male population sizes ranged from 1,418 (Table 2; 95 % CI = 1,335 - 1,582) to 4,107 (95 % CI = 2,790 – 6,551), which would represent 53-152 males per premises within the release block (Table 2).”

**Results, Figure 7, lines 395-397:**

**Were these estimates biased by not being able to tell males by the point of release? So long S -> N movement couldn’t be determined?**

Thank you for the comment. There is no statistical issue with inference for a multi-point release where the release location of the trapped individuals is unknown. If different coloured markings were used and this information were available, the dataset would be more informative and we would certainly expect to get better estimates, but it is certainly possible to perform the inference without this. In essence, our method treats the trapping intensity at each site as a finite mixture of the dispersal kernels from each release point, so the unknown latent variable of release location for each trapped mosquito has been integrated out of the probability density function used for the likelihood equation.

**Results, last paragraph, Lines 408-410.**

**Was this the same for unfed, blood fed and gravid females? Or do BGS traps collect mostly unfed females?**

We did not examine whether adults were unfed, blood fed or gravid. If anything, BGS traps have a slight bias towards collecting blood-fed parous females.

From the work of Ball et al 2010:

“Biases were not detected across age groups or body sizes. A significant bias was detected across physiological groups: nulliparous females were recaptured at a significantly lower rate than all other groups except blood-fed parous females, which were also recaptured at a low rate by the BGS.”

Ball, T. S., & Ritchie, S. R. (2010). Sampling biases of the BG-sentinel trap with respect to physiology, age, and body size of adult Aedes aegypti (Diptera: Culicidae). Journal of medical entomology, 47(4), 649-656.

**Discussion**

**Discussion, paragraph 1, line 415:**

**I’m not sure this generality relates well to Culex or even Anopheles where populations are larger and more widely distributed across the landscape?**

Thank you for the comment. This is a broad opening statement which we believe holds true considering the considerable investment in SIT for all genera of mosquitoes currently. We think it entirely depends on the spatial scale and the goal of control activities. The Chinese estimate their cost of producing Ae. albopictus males will be US$0.001ea this year. We see this and other techniques for sex separation as adaptable to Culex and Anopheles. The IAEA is undertaking SIT releases for Anopheles in Africa. Likewise SIT for Culex is being planned for avian malaria in Hawaii. And then there are gene drives and other genetic modification methods that will rely on inundative releases. To the authors of this paper, these opportunities represent a new great era for vector control.

Further to our point on population parameters, before any mass rearing can begin in these activities, it is important to understand the population size to ensure adequate “overflooding”. MRRs are the simplest and most efficient way to do this and are being recommended by the IAEA before any intervention to ensure mass-production is adequate.

**Discussion, paragraph 1, line 411:**

**This implies released males were less competitive for mates and required an over-flooding compensation?**

It is generally assumed that a Wolbachia infection has the minimum level of fitness costs for a sterile male program, generally around <20% for wALb and wMel. The relative competitiveness of sterile males can be assumed to affect the success of release programs in many ways which include; fitness cost of rearing (colony effects), sterilization and release method (stress), as well as things like the weather they are released into, the spatial distribution of releases relative to where the females are found (can males find the females?), and the number of background males in the wild population that sterile males need to compete with. Given that Wolbachia infected males should ideally have a fitness close to wild-type males before being released and that females generally only mate once, it can be assumed that competitiveness become female density (spatial coverage) and release frequency (temporal coverage) related variables. Because we released relatively similar numbers of marked males into different background populations the relative success of mating (competitiveness) will vary from experiment to experiment, as shown in the new competitiveness results.

**Discussion, paragraph 2:**

**Odd because males were 4 d old and should have been ready to mate a couple of days earlier….**

As the mating competitiveness results were not significantly different to wild-type males across all experiments, we have decided to remove this statement.

**Discussion, paragraph 2:**

**Odd – why should the proportion of females inseminated by wild males increase after marked males are released?**

We have removed this statement as this sentence was incorrectly worded and it was not relative to the narrative.

**Discussion, paragraph 2, lines 437-438:**

**Why not measure mating competitiveness?**

Thank you for the suggestion. On suggestion of reviewers we have included mating competitiveness.

“This result is unlikely to be influenced by trapping bias (56) or a lack of mating competitiveness by rhodamine B marked males.”

**Discussion, paragraph 2, lines 438-440:**

**Are these mostly unfed females? This seems very high!**

We were surprised by the daily proportion of unmated females and that is why we have drawn attention to it in the discussion. As we did not record whether females were fed or unfed, we cannot provide information on this status, only whether they had been mated or not. The short lifespan of *Ae. aegypti* males would suggest males would need to be regularly available to ensure mating, thus it is likely the species exhibits a high recruitment during optimal conditions. Results from the large IIT experiment run after this MRR work would suggest that releases 3 times a week are necessary to suppress a population to near-elimination.

Furthermore, BGS traps have a slight bias towards collecting blood-fed parous females. From the work of Ball et al 2010:

“Biases were not detected across age groups or body sizes. A significant bias was detected across physiological groups: nulliparous females were recaptured at a significantly lower rate than all other groups except blood-fed parous females, which were also recaptured at a low rate by the BGS.”

Ball, T. S., & Ritchie, S. R. (2010). Sampling biases of the BG-sentinel trap with respect to physiology, age, and body size of adult Aedes aegypti (Diptera: Culicidae). Journal of medical entomology, 47(4), 649-656.

**Discussion, paragraph 2, lines 444-445:**

**Unclear? Or do you mean emergence?**

We did mean emerge when using the phrase “receptive to mating”, however we have changed the phrase to “emerge” to use more precise language.

“Doing so will ensure a saturated landscape where incompatible males sterilize females as soon as they emerge while also minimising the effect of immigration.”

**Discussion, paragraph 2, lines 445-447:**

**Seems similar to normal larvaciding programs?**

We agree with this statement and have added it to the sentence.

“If sterile males are not constantly present within the landscape, then programs will tend to be inefficient with higher costs and lower suppression than expected over time, similar to normal larviciding programs.”

**Discussion, paragraph 3, lines 457-459:**

**Wouldn’t this depend on female movements after insemination?**

We believe this observation is correct and have added it to the sentence.

“This suggests marked males had an effect on the female population greater than the distance over which they were captured, likely due to female movement.”

**Discussion, paragraph 3, lines 464-469:**

**However, males mate at the host? So males would have the same host seeking requirement as females?**

The historic theory is that mating of *Ae. aegypti* in nature commonly takes place near the host; however, mating also commonly takes place away from the host around natural and artificial swarm markers. Unfortunately, there have been few studies to concretely confirm the when and where of *Ae. aegypti* mating and historic studies are often confounded by using human collectors.

In mating competition assays we have performed in semi-field cages females never had access to hosts, but we did place swarm markers in the experimental arena (e.g., large black buckets). Mating rates from these experiments was typically >90% suggesting that successful mating of large proportions of the female population is not reliant on host presence.

Furthermore, several studies demonstrate the transfer of male accessory gland proteins is required to increase female host-seeking and blood-feeding success. Based on this observation, one would have to assume that most females are mated in the absence of a host.

**Discussion, paragraph 5, line 489:**

**You have the opportunity to measure this here!**

Thank you for the suggestion to measure competitiveness. We have undertaken this analysis and included results and commented in discussion paragraph 2.

**Discussion, paragraph 5, lines 492-494:**

**Survival curves [exponential decay] always indicate sufficient numbers of females survive long periods.**

Thank you for the suggestion. We have included this wording into the sentence:

“It is estimated that female Ae. aegypti adults live on average for between five and nine days in the field, with survival curves showing enough females survive the extrinsic incubation period to transmit pathogens (13, 62-64).”

**Discussion, paragraph 5:**

**Experiments with Culex and Anopheles have indicated that treated males live as long as non-treated, but fail to mate competitively.**

We have removed our broad statement due to this complexity.

**Discussion, paragraph 6, lines 500-520**

**Differences in age structure?**

Following on from suggestions by reviewers to consider marked male mixing within the population and incorporating an aspect of survival, we have re-written conclusions related to the LPI. Below is the paragraph in which we incorporate the modifications to our findings.

“The LPI is a closed population method used historically for estimating population sizes during mark-release-recapture experiments. Ratios employed by this index compare the relationship between the total number of individuals captured, with the numbers released and then recaptured (8). Our results confirm the difficulty of estimating accurate population sizes with the LPI. These inaccuracies are most evident when we compare population estimates (Table 2) with the total male and female captures between seasons (Fig 4). The larger mean daily trapping rate during season 2 would suggest a population 1.5-2 times larger than during season 1. However, the LPI estimate does not reflect this trend, with results suggesting similar populations between MRR 3 and 4, but lower in MRR 5. Although inaccuracies in our population estimates are reflected by large confidence intervals, it is likely that even the lowest confidence intervals are unreflective of the male Ae. aegypti population in the field, which when divided by the number of premises within the release block suggest 53-75 males per house. Extensive Ae. aegypti research in north Queensland suggests this population estimate is much higher than would be expected in the region, given the number of adults typically observed per premises during the wet season is ca. 10 (65, 66). Furthermore, Ae. aegypti is an urban container inhabiting mosquito and populations are correlated with precipitation and temperature (65, 67). The lack of rainfall during the two weeks leading up to MRR 3, (0.8 mm) would suggest a considerably lower population when compared to MRR4 (242 mm). However, this was not the case when populations were estimated by the LPI and this could be the result of low re-captures. Future experiments that combine both the LPI and the insemination rate (or competitiveness) could overcome inaccuracies associated with low recapture rates and may lead to improved population estimates in the field”.

**Discussion, paragraph 6, lines 514-516:**

**But your insemination rate was still low indicating a high recruitment rate. Experiments with Culex and Anopheles indicate that released virgin females rarely remain virgins for >2 d….**

The insemination rate in our experiments may have been low indicating high recruitment, but this may be natural to the Ae. aegypti system or the north Queensland landscape as these findings have never been observed previously. It is likely that Ae. aegypti females are similar to Culex and Anopheles systems, with most females mated within two days.

**Discussion, paragraph 7, lines 527-530:**

**Or dispersal by females after insemination?**

We have included inseminated females moving into surrounding blocks.

“However, roads or wind direction did not totally prevent male movement or inseminated females moving into surrounding blocks, which suggests the open areas of Innisfail (such as roads or open grassy areas) only provided minimal resistance.”

**Discussion, paragraph 7, lines 530-535:**

**Could males ‘stack up’ at this barrier and be more frequently captured? Was this the same pattern as wild males?**

We believe it is unlikely that they were stacking up as this trap was in the middle of a forest line. Instead we think they may have been attracted to it as a dark harbourage area (see final sentence in paragraph). This extreme aggregation pattern was not observed in wild males, however, we did detect small numbers of male and female *Ae. aegypti* along forest lines.

“This observation supports previous studies that suggest that both male and female Ae. aegypti may instinctively move towards dark harbourage areas to seek shelter (68).”

**Discission, paragraph 8, lines 541-544:**

**Orientation problems finding resting sites?**

Thank you for your suggestion. We have included a sentence to reflect this potential reason for the movement patterns we observed.

“Alternatively, males may have had orientational problems finding resting sites, thus increasing movement across the landscape. Although we observed higher overall male movement during the night release, the lowest proportion of mated females during single-point releases was observed during this experiment.”

**Reviewer 2:**

**Introduction**

**Introduction, paragraph 3, lines 74-88:**

**It is worth pointing out here that the radius of the trapping area has strong effects on the distances travelled as shown in Guerra et al. (Ref #9), and the studies cited had relatively small radii.**

This paragraph has been re-written to incorporate the new results from references provided by the reviewer.

“Early movement studies suggest the majority of male Ae. aegypti disperse within 50 m of release sites after one week (12-16), although these estimates may be attributable to small radi in trapping arrays (9).”

**Introduction, paragraph 3, lines 79-83:**

**This comparison neglects recent work that showed similar movement of males and females and at longer distances with a more expansive spatial trapping array than the cited studies:**

**Marcantonio M, Reyes T, Barker CM. Quantifying Aedes aegypti dispersal in space and time: a modeling approach. Ecosphere. 2019;10. https://doi.org/10.1002/ecs2.2977**

**Juarez JG, Garcia-Luna S, Chaves LF, Carbajal E, Valdez E, Avila C, et al. Dispersal of female and male Aedes aegypti from discarded container habitats using a stable isotope mark-capture study design in South Texas. Sci Rep. 2020;10: 6803. doi:10.1038/s41598-020-63670-9**

We have incorporated these two modern studies into our reference list and now describe their movement results into the Introduction.

“Increasingly, studies have enlarged trapping distances and improved marking methods and statistical methods for estimating movement distances of both male and females Ae. aegypti (17-19). Recent dispersal studies suggest that males move further than females, with mean distances travelled (MDT) of between 196 m and 294 m over experimental periods (17-19).”

**Introduction, paragraph 3, lines 81-84:**

**what is meant by "rapidly"?**

We have removed rapidly and changed the sentence to represent the greater movement distance of *Ae. aegypti* males compared to females and provided references.

“Recent dispersal studies suggest that males move further than females, with mean distances travelled (MDT) of between 196 m and 294 m over experimental periods (17-19).”

**Introduction, paragraph 4, line 89:
Suggest changing "adequate population over-flooding" to something like "adequate release numbers to supplant matings with wild males"**

We have changed the sentence to reflect this description. However, the use of “over-flooding ratio” has been used since the early days of SIT (see Knipling 1952). We believe it is necessary to introduce and use this term when describing the challenge of estimating population sizes and how MRRs can assist in determining important biological parameters when estimating over-flooding ratios.

New sentence:

“The primary challenge for SIT/IIT strategies is the determination of adequate release numbers to supplant matings with wild males (or “over-flooding ratio”) across large areas.”

**Introduction, paragraph 4, lines 98-100:**

**and see earlier comment on the paper by Marcantonio et al. that utilized dispersal kernels**

We have added this reference to the sentence:

“More recently, mosquito movement studies have incorporated dispersal kernel theory, where distributions of movement can be estimated over the entire flight range (18, 23) by integrating a temporal component such as average life expectancy (19).”

**Introduction, paragraph 5, line 114:**

**should be excited?**

We have changed to word to “excited”

**Introduction, paragraph 5, line 114:**

**statement is accurate, but from conversations with other scientists who have done mark-recapture with rhodamine, this number seems like the best-case scenario and may not hold as a typical value.**

We thank the reviewer for their comment on the efficacy of rhodamine B as a mark. It is from our considerable experience with this mark that ~95% marking efficiency was our observed through multiple, well-replicated and controlled experiments. However, as with all methods a degree of experimental variation is expected. This variation could be attributable to differences in the strain of *Ae. aegypti* used, rearing and holding conditions, and differences in the control of female age or age at male introduction, and the training of staff and quality of equipment used in the detection and scoring of Rhodamine B marking. We acknowledge that the percent of successful transfer in select cases may be lower than 95%, but we cannot substantiate these observations through our own experience.

**Methods**

**Methods, paragraph 2, lines 149-153:**

**There is strong selection during initial colony establishment that can lead to biased MRR results with colony mosquitoes. Supplementation with wild mosquitoes will help diversity, but it would be useful to report the total time in colony prior to MRR and ideally the approximate range of generations represented in the MRR from the earliest colony establishment to the last addition of field-collected eggs.**

We have amended the paragraph to include the generations within each colony before experimental releases were undertaken:

*“*Aedes aegypti colonies were newly established with wild type eggs collected from multiple ovitraps in Innisfail before each experimental period. Mosquito colonies were maintained using standard laboratory rearing protocols with 28 °C ± 1 °C, a 70 % (± 10 %) relative humidity, and a 12:12 hour light cycle and twilight period. F1 – F3 generation wild-type Ae. aegypti larvae were hatched into a solution of 0.2 g/L yeast in water in which they were allowed to feed for 24 hours.”

**Methods, paragraph 3, lines 170-171:**

**indicate what is meant by a multi-point release**

We have provided further detail on what is meant by a multi-point release and indicated on which figure this design is displayed:

“For multi-point releases, males were divided evenly and released at five points along the eastern side of the central road (MRR 3 & 5, Fig. 1A).”

**Methods, paragraph 5, lines 183-184:**

**indicate the length of time the traps were operated post-release for each study oor criteria for termination of trapping if the end is data-dependent. In the latter case, the trapping periods for each study should be indicated also in the results.**

We have indicated the criteria for traps to cease operating as the day when no males contained rhodamine B.

“All traps were serviced daily throughout each MRR experiment until no marked males were present in the sample.”

**Methods, paragraph 6, Lines 191-193:**

**apologies if I missed it, but I did not see PDS defined anywhere above.**

We had not defined this previously and have now done so in the text.

“For all experiments the probability of daily survival (PDS) was estimated by regressing log10 (x+1) the number of recaptured males against days since release, where the antilog10 of the regression slope was the PDS (39).”

**Methods, paragraph 7, lines 201-207:**

**no study fully "meets" these criteria. Instead, any slight violations are presumed small enough to be ignorable, so I would say something like "were met with reasonable certainty"**

Thank you for the correction to our language. We have had to modify the way the LPI was implemented in our analysis on the suggestion of another reviewer. We have re-written the paragraph and the wording to reflect the reviewer’s suggestion:

“To account for the low survival of released Ae. aegypti males, only marked males captured the days before Tthe average life expectancy ALE (rounded to the nearest whole number) of each experiment waeres usedselected. to estimate Pmale Ae. aegypti populations for MRR 3, 4 and 5 estimates, where applicable, were calculated viausing the LPI with the Chapman modifier (41) within the primary release block:

N ̂=× n_2 ÷m_2 N ̂=((n_1+1) 〖(n〗_2+1))/((m_2+1))-1

Where N ̂ is the size of the population, n_1 is the number of marked animals released into the population, n_2 the total number of individuals captured (marked and unmarked), and m_2 the total number of marked individuals recaptured. All fiveThe remaining assumptions of the LPI (8) were met with reasonable certainty (8) which include: 1) the mark should not affect insects, 2) marked insects are allowed to become completely mixed with the local population, 32) sampling is random with respect to marked insects, 43) samples are measured at discrete time intervals in relation to total time, and 54) the population is not unduly influenced by immigration or emigration during the period of study.”

**Methods, paragraph 7, line 199:**

**how was this determined? (the mark should not affect insects)**

It was assumed with reasonable certainty that the rhodamine B had minimal effect on marked males. This assumption is supported by the experimental work by Johnson et al 2017, which showed the rhodamine B mark has minimal effect on wild-type and wMel *Ae. aegypti* male survival and mating competitiveness under semi-field conditions.

Please see:

Johnson BJ, Mitchell SN, Paton CJ, Stevenson J, Staunton KM, Snoad N, et al. Use of rhodamine B to mark the body and seminal fluid of male Aedes aegypti for mark-release-recapture experiments and estimating efficacy of sterile male releases. PLoS neglected tropical diseases. 2017;11(9):e0005902

**Methods, paragraph 9, lines 224-225:**

**For code and data, ensure that these links are permanent. I know Github links can be modified over time, so it would be better to provide the code as a supplement or to place it in a suitable repository with DOI.**

We have stored and provided permanent DOIs for the STEIK R code and raw mosquito trap data within the CSIRO Software and Data Collections, respectively. These collections form part of the Data Access Portal.

The Commonwealth Scientific and Industrial Research Organisation (CSIRO) was created in 2016 and is Australia's national science agency, one of the largest and most diverse research agencies in the world. CSIRO is committed to ensuring its online content is accessible to everyone regardless of ability. The CSIRO Data Access Portal (DAP) provides access to research data, software and other digital assets published by CSIRO across a range of disciplines. The DAP is maintained by CSIRO Information Management & Technology and is a permanent archive for the preservation of research data assets retained for reference and reuse purposes.

**Methods, paragraph 10, lines 231-250:**

**include citations and versions for R and the packages used.**

We have included citations for all R packages used. The R version used is included.

“The R package ‘glmmTMB’ (46) was used for all mixed effects models and the packages ‘DHARMa’ (47) was used for model diagnostics and ‘ggplot2’ (48) for visualisations. To look for collinearity in predictors, correlations were examined using the R package ‘corrplot.’ (49). The Wall-Raff rank sum test of angular distance from the R package ‘circular’ was used to compare similarities between wind direction and male recapture angle (50). To compare whether rhodamine B and wild males and wild female Ae. aegypti were more likely to be captured by BGS traps at certain locations (house, backyard or forest), we used contingency table analysis with odds ratios calculated via the R package ‘epitools’ (51). All analyses were performed using R version 3.5.3 (52).”

**Results**

**Results, paragraph 1, lines 271-274:**

**meaning is unclear - how was this determined? why not test this with the model? the assumption of isotropy is a strong one, especially if based on cursory visual inspection.**

To support our observation, we have included an analysis that applies circular statistics to compare the average wind direction and average mosquito movement angle from the release point for each season.

“Wind direction was predominantly from southeast to north east (Fig. S2) and was significantly different to the daily mean angle of movement for male *Ae. aegypti* from single point releases from season 1 (ꭓ^2^ = 9.05, *P* < 0.005) and season 2 (ꭓ^2^ = 12.06, *P* < 0.001) (Figures S3).”

**Results, paragraph 2, line 280:**

**seems to deviate from the season 1 and 2 designations - are these the same thing? need consistency for the reader to follow.**

We have now defined season 1 and season 2 as “late dry” and “wet” season earlier in the methods.

“Mark-release-recapture experiments 1-3 (season 1) occurred late dry season, between the 18^th^ November and 13^th^ of December 2016, while MRR experiments 4-6 (season 2) occurred during the wet season, between the 7^th^ and 27^th^ of February 2017.”

**Results, paragraph 2, line 284:**

**keep nomenclature consistent throughout - seasons were indicated with numerals 1 and 2 above.**

We have ensured nomenclature for seasons are consistent here and throughout.

**Results, paragraph 3, lines 299-303:**

**These R^2 values seem extremely high. Is this just due to having a small amount of data? What is the correlation being expressed?**

We have removed the R^2^ values as they not necessary for a probability of daily survival.

**Results, paragraph 4, lines 316-319:**

**strikes me as unexpected - what is a plausible reason for this?**

There is a lot of random variation associated with all six experiments that we believe explains results such as this. We also expect, as discussed in the discussion, that there were differences in the wild population size between late dry and wet seasons which would influence the effectiveness of released males, best described as the overflooding ratio. Here we released the same number of males into different wild population sizes across two seasons, and this was reflected in competitiveness and therefore the proportion of wild females mated. When taken by itself however, competitiveness of marked males did not vary significantly from wild males.

**Discussion**

**Discussion, paragraph 4, lines 459-472:**

**In this paragraph and the paragraph above on dispersal distances, it would be interesting to contrast this study's findings and spatio-temporal modelling approach with those in the similar Marcantonio et al. study with stable isotopes mentioned in my comments on the introduction.**

Thank you for your suggestion which we think has improved our comparison with recent literature. We have incorporated both the similarities in MDT between studies and differences in kernel frameworks into paragraphs 4 and 5 in the discussion.

**Paragraph 3:**

"Our results support recent studies which show male Ae. aegypti are capable of moving further through an urban landscape than historical observations suggest. Variations in MDT are likely due to the chosen mark, collection method and landscape configuration (12) and results have implications for how, where and when mosquito programs release males during SIT/IIT interventions into local landscapes. Here we show that male Ae. aegypti have the potential for long distance movement despite a short lifespan, supporting recent dispersal studies using stable isotopes (17-19, 21). During single point releases marked males were recaptured at distances greater than 350 m and 400 m after day one and two, respectively, suggesting rapid movement when compared to females of the species. Furthermore, rhodamine B inseminated females were captured at the extent of the trapping array and we observed a MID greater than the male MDT. This suggests marked males had an effect on the female population greater than the distance over which they were captured, likely due to female movement. Both isotropic and traditional MDT measurements of male movement (between 95 m and 462 m and across movement barriers) support the general observation that the rate of male movement was greater than those traditionally recorded for females of the species in Australian landscapes. This could suggest that gene flow within an isolated population may be related to male spread as female movement is typically observed at less than 100 m over a lifetime (13, 57). This difference may be due to the different biological requirements of each sex, with females requiring a blood source, a resting place and oviposition site that may reduce dispersal, while the short life expectancy of males may have them constantly searching for virgin females. A greater understanding of dispersal patterns in local environments will result in maximum sterile male coverage across a landscape, and lead to greater mating success, the primary goal of area-wide release programs.”

**Paragraph 4:**

“While single point MRRs are ideal for measuring distance travelled over time, the utilization of measurements from multi-point releases are essential to optimizing area-wide releases. The STEIK estimates of movement used in this study (19, 42) benefited from the addition of temporal data and tended to produce larger estimates than those calculated through traditional methods. Interestingly, our kernel estimates of male MDT were similar to Marcantonio et al (18) over the period of one week. However, static kernels such as this and Winskill et al (23) are based on seed dispersal where individual movement is not estimated once collected in traps (19). The STEIK method has the additional benefit of estimating movement throughout a life-time as a temporally evolving kernel.”

**Discussion, paragraph 4, lines 470-477:**

**please clarify - wouldn't it be helpful if models acknowledge losses due to trap captures?**

Accounting for mosquito catches in traps would undermine the simplicity of using the Gaussian diffusion model in the STEIK framework to infer movement parameters. This is an assumption of the model and as with most assumptions, including this one, they can lead to inaccuracies. We make this assumption to allow the system to be modelled very simply.  If recapture rates were to be very high, then this would need to be accounted for in the analysis, but this would most likely then require a much more complex method of modelling and analysis.

This statement has been removed as we rewrote the section:

“While single point MRRs are ideal for measuring distance travelled over time, the utilization of measurements from multi-point releases are essential to optimizing area-wide releases. The STEIK estimates of movement used in this study (19, 42) benefited from the addition of temporal data and tended to produce larger estimates than those calculated through traditional methods. Interestingly, our kernel estimates of male MDT were similar to Marcantonio et al (18) over the period of one week. However, static kernels such as this and Winskill et al (23) are based on seed dispersal where individual movement is not estimated once collected in traps (19). The STEIK method has the additional benefit of estimating movement throughout a life-time as a temporally evolving kernel.”

**Discussion, paragraph 5, line 484:**

**there are other studies outside Australia that should be referenced on survival of aegypti in the field. See for example, the following model-based synthesis by Brady et al:**

Thank you for the suggestion. We have included further studies relevant to the survival of female Ae. aegypti.

**Discussion, paragraph 6, lines 511-513:**

**what is the basis for believing one vs another here?**

For a number of decades, we have studied populations of Aedes aegypti extensively in north Queensland. Our observations and measurements suggest that populations of the species are generally not as high as other tropical locations, likely due to a number of social and climatic reasons. We have modified the sentence to suggest that in our extensive experience the dry season population is unlikely to be this high and have provided references to support this.

“Extensive Ae. aegypti research in north Queensland suggests this population estimate is much higher than would be expected in the region, given the number of adults typically observed per premises during the wet season is ca. 10 (65, 66).”

We give further justification later in the paragraph (**Lines 515-516**):

“The lack of rainfall leading up to MRRs 1 and 2, when compared to season 2 would suggest a lower population leading up to and during the dry season experiments.”

**Discussion, paragraph 7, lines 521-522:**

**as above, see Marcantonio et al. and Juarez et al., which did study male movement, although clearly more study is needed as the authors point out.**

We have changed this sentence to reflect the contemporary studies.

“Until recently, a lack of male Ae. aegypti movement studies resulted in a deficiency of knowledge on individual behaviour and their ability to navigate through landscapes.”

**Discussion, paragraph 8, lines 537-528:**

**or possible that a night release would simply allow more time for movement before achieving initial mating success? It is not clear how mating might affect future movement (e.g., could males move less once they have found a location where they were successful in finding a mate?)**

Thank you for the alternative hypothesis. We have added this suggestion to the text.

“However, when males were released at night, we observed higher recaptures, a greater total MDT and a rapid mixing within the landscape when compared with daytime releases. It is possible that *Ae. aegypti* males move further through the night with low predation, higher humidity and lower predation playing less of a role than during daylight. Alternatively, males may have had orientational problems finding resting sites, thus increasing movement across the landscape.
