# Supplementary material for: Mark-release-recapture of male Aedes aegypti (Diptera: Culicidae): Use of rhodamine B to estimate movement, mating and population parameters in preparation for an incompatible male program
Source: PLoS Negl Trop Dis. 2021 Jun 7;15(6):e0009357. doi: 10.1371/journal.pntd.0009357 (PMC8183986; doi:10.1371/journal.pntd.0009357)
Supplement: Attachment — Submitted filename: Response to reviewers-Resubmit03.docx [file pntd.0009357.s009.docx]

**Response to reviewers**

We would like to thank the reviewer for the time they have spent providing further feedback on the current version of the manuscript. We have corrected the order of the Figures throughout the manuscript and fixed the referencing formatting errors.

**Lines 191-193: My previous comment was misinterpreted: My question was meant to inquire was log10 was used for PDS and loge was used for ALE? Should these have been the same?**

These should not be the same. The calculation of the PDS in the literature has always calculated as the slope of a simple linear regression of the log_10_ transformed number of mosquitoes recaptured or surviving over time. The calculation for ALE in the literature has always been the antilog of the slope which is the natural log of the PDS.

Please see:

Niebylski M, Craig Jr G. Dispersal and survival of *Aedes albopictus* at a scrap tire yard in Missouri. J Am Mosq Control Assoc. 1994;10(3):339-43.

Johnson, B. J., Hugo, L. E., Churcher, T. S., Ong, O. T., & Devine, G. J. (2020). Mosquito age grading and vector-control programmes. *Trends in parasitology*, *36*(1), 39-51.

Ndoen, E., Wild, C., Dale, P., Sipe, N., & Dale, M. (2012). Mosquito longevity, vector capacity, and malaria incidence in West Timor and Central Java, Indonesia. *International Scholarly Research Notices*, *2012*.

David, M. R., Lourenço-de-Oliveira, R., & Freitas, R. M. D. (2009). Container productivity, daily survival rates and dispersal of Aedes aegypti mosquitoes in a high income dengue epidemic neighbourhood of Rio de Janeiro: presumed influence of differential urban structure on mosquito biology. *Memórias do Instituto Oswaldo Cruz*, *104*(6), 927-932.

Harrington, L. C., Buonaccorsi, J. P., Edman, J. D., Costero, A., Kittayapong, P., Clark, G. G., & Scott, T. W. (2001). Analysis of survival of young and old Aedes aegypti (Diptera: Culicidae) from Puerto Rico and Thailand. *Journal of medical entomology*, *38*(4), 537-547.

**Lines 201-202: So this method presumes that no released males died until the average length of life? The assumption of ‘immigration’ is really additions which would include emergence. Because the wild females had such a low insemination rate [60%], the emergence of addition rate must have been elevated?**

The reviewers have drawn attention to a number of problems when applying the Lincoln Peterson Index to a mosquito system. In this manuscript we discuss problems associated with the LPI and have also discussed these directly with the reviewers. Like all models, there are a number of assumptions that need to be made which will may affect the accuracy of results These assumptions relate to the lifespan of mosquitoes and the flux of individuals entering or leaving the study area (i.e. how closed the population is). The validity of these assumptions will vary from experiment to experiment. With mosquito systems, assumptions of closed populations may be flawed, but immigration and emigration are very difficult to quantify and therefore simplifying assumptions must be made and calculations from models are based on these. When new data becomes available, such as the insemination rate observed in this study, it can have the effect of drawing attention to the failures of this particular model (the LPI) in the system being studied. In this case, our results cannot be used to invalidate all previously studies that did not observe an insemination rate, but instead we can draw attention to this issue, so that future estimates take this into consideration. We have included extra information to explain this in the discussion:

**Lines 488-498:** “*Ratios employed by this index compare a simplistic relationship between the total number of individuals captured, with the numbers released and then recaptured (8). Our results confirm the difficulty of using this index to accurately estimate mosquito population sizes, particularly due to a lack of biological information during releases. For instance, it is enforce the strict almost certain that the assumption of a closed mosquito population closed and that the probability of recapture remains constant over space and time are incorrect. This is because new individuals are constantly being recruited into a population during favourable periods but are also dying constantly due to short lifespans, in heterogeneous spatial distributions across a landscape. When we observe proxies for these rates, such as estimates of average lifespan and mating rates, our faith in the estimates obtained from the LPI may diminish, depending upon to what extent the above assumptions may be violated.”*

and

**Lines 513-515:** “*Future experiments that combine both the LPI and the insemination rate (or competitiveness) could overcome inaccuracies associated with low recapture rates and may lead to improved population estimates in the field*.”

The modification we have made to our calculations was done to respond to review comments around how closed the population was to immigration and emigration. Our rationale for limiting the dataset to only a small number of days after release was twofold: (i) this ensures that the LPI calculation is robustified to the influence of low recapture rates towards the end of an MRR (when most of the released males are dead) and reduces the influence of demographic stochasticity on the estimate; and (ii) over shorter windows of time, the amount of immigration, emigration, births and deaths in the study area is reduced. In this way, the underlying assumption is that births, deaths, immigration and emigration are in balance, so that the population of unmarked individuals remains stable. In addition, we only included those males captured in the release block, which is surrounded by large movement barriers to remove the bias associated by these barriers in our experimental design.

**Line 419: Did not seem to be the case in Fig. 4?**

While the trend was hard to observe in Figure 4, there were increases in the proportion of total females mated from day 1 to day 2 in 4 out of 4 experiments, increases from day 2 to day 3 in 4 out of 6 experiments and increases from day 1 to day 3 in 3 out of 4 experiments. However, these did were not significant in most cases, just a trend in the data. For simplicity we have decided to remove the statement on line 419 from the discussion.

**Lines 417-420:** “*Marked males showed no significant differences in mating competitiveness across experiments. This made no difference to the proportion of all inseminated females throughout each experiment (both rhodamine B and wild mated), and this effect was consistent across seasons.*”

**Line 454: But they congregate to mate at the host so males would be expected to have the same attractions as females?**

The historic theory is that *Ae. aegypti* mating commonly takes place near the host. This observation is an inherent human bias where an observer can act as a swarm marker. Because of this the host is commonly thought to be primarily where females are observed mating. We know that mating does occur away from humans, and our observations suggests males are found away from humans in their movement. We observed males in areas of forest when there were no human hosts. Females were also observed in these forested areas. Our results suggest that males do not necessarily aggregate or move through the same landscape features (as females would) when searching for mates. Hence, our observation of increased rate of movement and total movement of males. We have also observed that mating commonly takes place away from the host around natural and artificial swarm markers. Unfortunately, there have been few studies to concretely confirm the when and where of *Ae. aegypti* mating and historic studies are often confounded by using human collectors.

In mating competition assays we have performed in semi-field cages females never had access to hosts, but we did place swarm markers in the experimental arena (e.g., large black buckets). Mating rates from these experiments was typically >90% suggesting that successful mating of large proportions of the female population is not reliant on host presence. Furthermore, several studies demonstrate the transfer of male accessory gland proteins is required to increase female host-seeking and blood-feeding success. Based on this observation, one would have to assume that most females are mated in the absence of a host and therefore not have the same level of attractiveness.
